# Influence of thickness and strength on plastic instability in tailored steel structures

**DOI:** 10.1038/s41598-024-66331-3

**Published:** 2024-07-04

**Authors:** Rihuan Lu, Shoudong Chen, Xiaogong Wang, Meihui Li, Sijia Zhang, Sai Wang, Xianlei Hu, Jingqi Chen, Huagui Huang, Xianghua Liu

**Affiliations:** 1 National Engineering Research Center for Equipment and Technology of Cold Rolled Strip, Yanshan University, Qinhuangdao, 066004 Hebei People’s Republic of China; 2 School of Mechanical Engineering, Yanshan University, Qinhuangdao, 066004 Hebei People’s Republic of China; 3 School of Mechanical Engineering, Tongling University, Tongling, 244061 Anhui People’s Republic of China; 4 School of Metallurgy and Energy, North China University of Science and Technology, Tangshan, 063210 Hebei People’s Republic of China; 5 Northeast Refining and Chemical Engineering Limited Liability Company, Chinese Petroleum Group Company, Shenyang, 110015 Liaoning People’s Republic of China; 6 The State Key Laboratory of Rolling & Automation, Northeastern University, Shenyang, 110819 People’s Republic of China; 7 Research Institute of Interdisciplinary Innovation, Zhejiang Lab, Hangzhou, 311121 People’s Republic of China

**Keywords:** Tailor rolled blanks, Tailor rolled tubes, Plastic instability, Equivalent strength, Loading, Engineering, Materials science

## Abstract

A mathematical model was intricately devised to explore the influence of continuous variations in thickness and mechanical properties on the performance of tailor rolled blanks (TRB) and tailor rolled tubes (TRT). Through the integration of analytical and numerical techniques, it was discerned that these variations play a pivotal role in modulating stress distribution and strain localization, thereby inducing a spectrum of plastic instability behaviors within the structures. The introduction of an ‘equivalent strength’ metric as a novel means to quantify structural performance shed light on strategic material distribution to enhance durability and mechanical efficiency. Moreover, the insights garnered from this research deepen the understanding of the mechanical responses of tailor-rolled constructs under varying loads, offering valuable perspectives for the development and fabrication of engineered materials with bespoke properties. This study not only contributes to bridging a knowledge gap in the realm of tailored material engineering but also fosters the advancement of design methodologies in the construction of high-performance engineered structures.

## Introduction

In engineering applications, variable-thickness components, due to non-uniform force distribution, offer better material optimization than uniform ones. Strategic thinning can enhance lightweighting and crashworthiness. Tailored blanks have been developed by rolling partially stacked blanks, focusing on thickness variations^1^. Bi-directional thickness can be achieved through methods like “constant roll gap process” and “variable roll gap process”^2^. A twin-roll casting method was introduced, optimizing parameters such as plastic strain and exit-strip temperature, to produce varied-width aluminum sheets^3^. A mobile electromagnetic edge-dam aids in pre-solidification metal containment, enabling near step-like width changes. However, issues like control accuracy and production efficiency remain unresolved. In the engineering domain, TRBs fabricated through Variable Gauge Rolling (VGR) have gained prominence. Thickness adjustments during rolling are facilitated by hydraulic cylinders, governed by computer algorithms. Techniques for variable thickness rolling have been rigorously examined. Investigations into Flying Gauge Change (FGC), emphasizing roll gap and speed, have been conducted^4^. The use of variable thickness to generate configurations, notably the “dog-bone” profile, has been observed^5^. A multi-point setting methodology for thickness control has been introduced^6^. Distinct methodologies for TRB production, focusing on longitudinal and latitudinal transitions, have been elucidated^7^. Continuous thickness transition zones in TRBs enhance load adaptability. Principles of force equilibrium and mass conservation have informed tailor rolling. A dedicated TRB production line has been established, augmenting engineering diversification^8^. Aluminum strips with lateral thickness variations have been produced using specialized roll profiles^9^. Metal flow during rolling and stamping significantly influences thickness variations. In summation, efficiently fabricated TRBs offer precise thickness customization with controllable transition zones.

The rising significance of TRBs in engineering production has been emphasized, with these becoming essential for fabricating components like beams and frames^10^. High-pressure sheet metal forming, enhanced by simulation and optimization, is commonly used to produce TRBs. Highlighting the benefits of dynamic roll gap adjustment, TRBs have been described as innovative products with superior deep-drawing capabilities^11^. Integrating VGR with suitable forming techniques results in precise TRBs. Given that engineering parts often serve as energy absorbers, significant research focuses on producing hollow tubes with variable thickness. Addressing Tailor Welded Tubes (TWT) limitations, methods like tailor layered tube hydroforming have been adopted^12^. Specialized adjustable tools have been introduced for intricate structures^13^. Techniques like Wire-cut Electrical Discharge Machining (WEDM) have been employed for thin-walled square tubes^14^. Forward extrusion, with an adjustable bearing location, has been effectively used for AA6060 aluminum tubes^15^. Multi-stage deep drawing optimizes load and deformation in cylindrical tubes^16^. In conclusion, while challenges persist in producing variable thickness structures, combining VGR with subsequent forming techniques remains the leading production method for tailor-made tubes.

Localized mechanical property tailoring, combined with thickness optimization, can elevate vehicle performance and crashworthiness. In material engineering, specialized furnaces have been used for localized blank heating^17^. Techniques for optimizing mechanical properties in blanks for hot stamping through localized heating have been explored^18^. Tailored tempering processes have been applied to adjust temperature distribution in blanks^19^. By leveraging thermal insulating materials, blanks with region-specific mechanical properties were crafted. Local resistance heating was employed to create variable hardness zones in steel blanks^20^. Selective laser heat treatment yielded steel blanks with distinct hardness gradients^21^. Induction and conduction heating, guided by Finite Element Modeling (FEM), were integrated for zonal heating of billets^22^. The introduction of Local Induction Heating Bending Forming (LIHBF) technology addressed challenges in thin-walled steel tubes^23^. Laser heat treatment on crash boxes enhanced energy absorption during axial collapse^24^. Techniques like inductive heating post-roll forming were used for tubes with tailored properties^25^. Overall, local induction heating and laser treatments show promise for segmenting properties in thin-walled structures, highlighting their potential for future advancements. Engineering components benefit from adjustments in both thickness and mechanical properties to optimize load capacity and lightweight design. Dual-phase steel TRBs (TRB-DP) exhibited varied mechanical properties post-annealing due to prior rolling hardening^26^. Studies on medium-manganese steel TRBs during annealing revealed differences in tensile strength across thickness zones, influenced by annealing temperature^27^. A two-step overaging process was introduced for TRB-DP, leading to noticeable changes in mechanical properties^28^. Comprehensive evaluations of annealing methods showed that TRB properties can be modulated through specific parameters^29^. In summary, heat treatments like annealing and overaging allow precise tailoring of TRB properties, enabling control over component thickness and mechanical attributes.

Global research has addressed structural instability in thin plates and thin-walled structures, emphasizing their elastic–plastic buckling. Analyzing these phenomena is crucial for predicting buckling sites, deformation paths, and limit loads. Recent advancements include structural optimization, artificial geometric defects, and external device-induced deformations to enhance thin-walled structures’ performance. Using Finite Element Modeling (FEM) and experimental methods, the plasticity and failure behavior in steel sheets and thin-walled tubes were explored^30^. Buckling behavior in functionally graded (FG) cylindrical shells was analyzed using first-order shear deformation theory^31^. External inversion molds were employed to study deformation mechanisms in Functionally Graded Thickness (FGT) tubes^32^. Innovative grooving patterns in thin tubes revealed their impact on initial buckling deformation^33^. Alternative methods, such as a chamfer external trigger, provided insights for energy-absorbing braided tube designs^34^. The modified Bidirectional Evolutionary Structural Optimization (BESO) algorithm optimized the material layout of thin-walled square tubes^35^. Origami patterns applied to various tubes enhanced their performance, and experimental studies on graphite/epoxy laminate tubes highlighted the effectiveness of the chamfer failure trigger^36,37^. In conclusion, by adjusting geometrical properties and external conditions, the performance of thin-walled structures can be optimized.

In advanced manufacturing and heat treatment, the integration of VGR with modified treatments is pivotal for producing tailored components with adjustable thickness and properties. VGR allows for continuous TRB production with customized thickness, while innovative methods like local induction heating and selective laser heat treatment are emerging for nuanced heat treatments. This research examines the structural behavior of tailored components under varied loads, focusing on their mechanical properties during uniaxial tension and buckling during axial compression. TRB samples, crafted using VGR, underwent specific heat treatments based on calculated austenitic phase transition temperatures (AC1). The resulting microstructural changes were analyzed in depth. Both experimental and computational methods were used to study the deformation and buckling of specimens derived from TRBs. The concept of ‘equivalent strength’ was introduced, linking deformation patterns, mechanical reactions, and material property distributions. The mechanical behaviors of these specialized components were then compared to conventional ones, ensuring weight and material strength consistency.

### Specimen preparation and fabricating process

#### Preparation of experimental tailor structures

**Figure 1:**
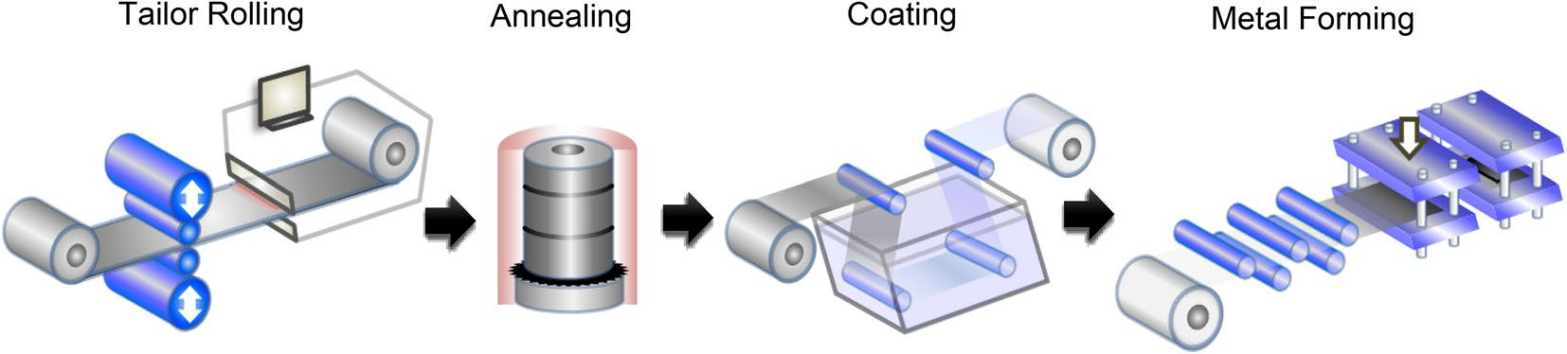
Technological process of cold formed TRB-parts.

The technological workflow for fabricating TRBs is depicted in Fig. 1. Initially, dynamic variable roll-gap control is executed on a cold rolling mill equipped with a rapid hydraulic reduction mechanism. By judiciously synchronizing the rolling speed in the lateral dimension with the reduction speed in the vertical plane, precise control over the length of regions with varied thickness, as well as the dimensions of the transitional zones, is achieved. In-line thickness gauges situated between the rolling mill and the coiler provide real-time measurements of TRB thickness. Any deviations from the target thickness are automatically corrected to ensure stringent thickness tolerances. This rolling operation is conducted in a cyclic and continuous manner until a TRB coil is fully processed. Subsequent to rolling, the coil undergoes recrystallization annealing in accordance with the stipulated mechanical property specifications. For certain materials, protective coatings are applied to the TRB’s external surface. The final TRB components are typically realized through press-forming operations.

**Figure 2:**
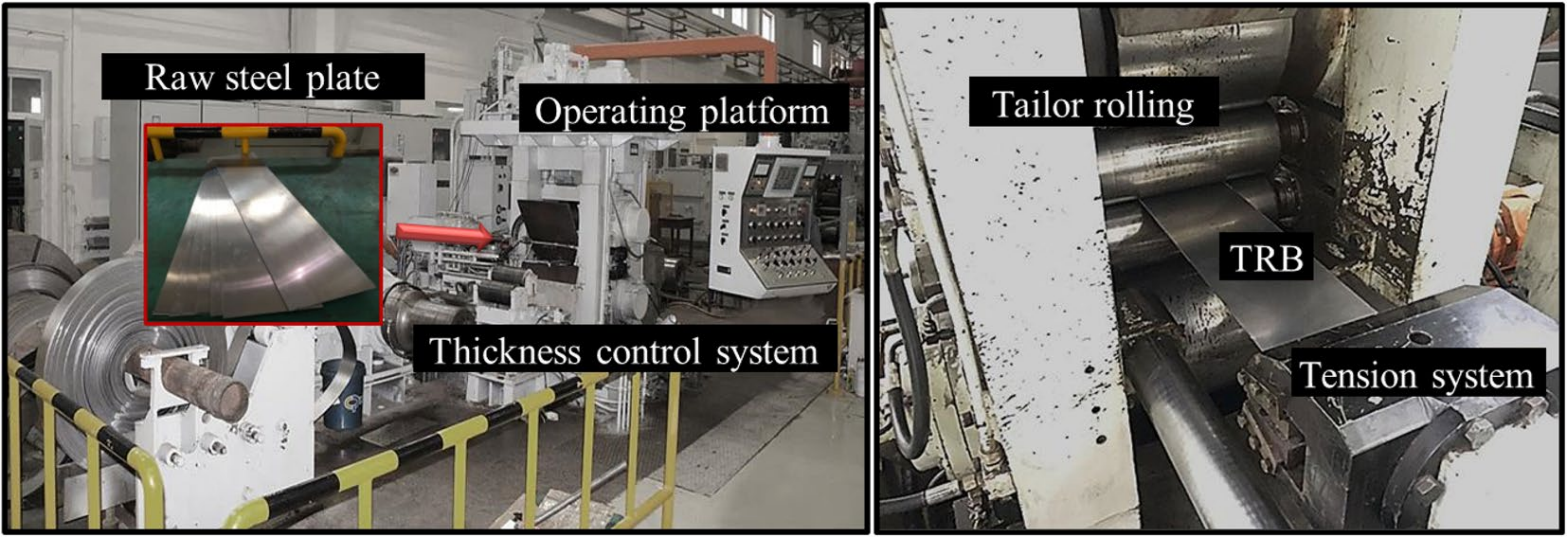
Experimental equipment of variable gauge rolling.

The 150 mm 4-high reversing mill is selected as the specialized production mill for TRBs, as illustrated in Fig. 2. The equipment selection for VGR primarily includes the mill roll, mill frame, main motor, Automatic Generation Control (AGC) hydraulic system, and crimping system. Given that TRB is predominantly utilized in the engineering industry, its thickness generally ranges from 1.0 to 3.0 mm. Owing to engineering strength requirements, the minimum sheet thickness is conventionally above 1 mm. Given the incoming sheet metal’s thinness and the limited absolute reduction post-rolling, a full hydraulic screwdown mechanism is employed, congruent with VGR characteristics. The rolling schedule primarily consists of single-pass rolling, with a thickness reduction often exceeding 50%. This imposes specialized requirements on the rolling mill equipment, necessitating heightened performance from the hydraulic and main motor drive systems. Distinctively, the automatic control system of VGR aims to achieve concurrent high-precision control of both length and thickness during rolling. To accomplish this, the system integrates features like high-precision blank rolling micro-tracking, variable rolling speed control, variable thickness control, and variable tension control.

**Figure 3:**
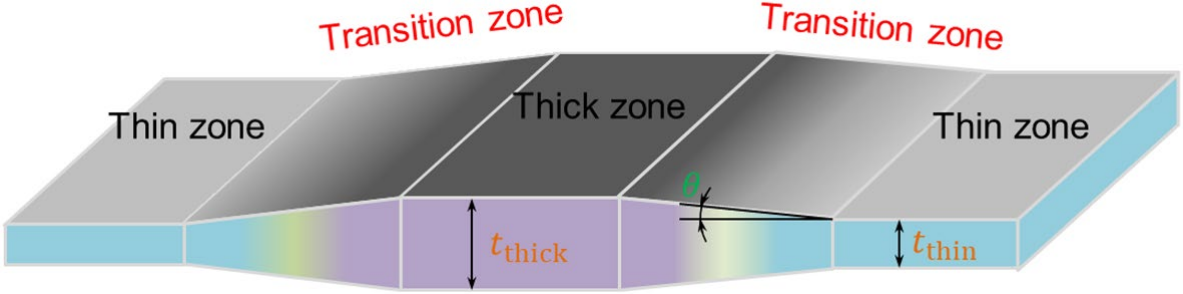
Schematic diagram of the periodic longitudinal TRB.

Produced via the VGR technique, TRBs typically exhibit a thickness range of 0.5 mm to 3.0 mm and are primarily employed in the engineering sector to achieve weight reduction. As delineated in Fig. 3, TRBs can be categorized into three distinct zones: thin, thick, and transitional. The transitional zone, situated between the thin and thick zones, exhibits characteristics governed by the interplay between roll gap alterations and rolling speed. To cater to the demands for geometrical flexibility, VGR rolling schedules can be manipulated to fine-tune the dimensions and thickness ratios of these zones. It is noteworthy that the current state of technology imposes a limit on the differential thickness ratio, which should not exceed a 1:50 ratio. Given the intricate load distributions often encountered in engineering applications, uniform thickness structures frequently fail to provide optimal load-bearing capabilities. TRBs, by virtue of their engineered thickness distributions, allow for a more congruent alignment between structural attributes and external loading conditions, thereby optimizing both performance and weight efficiency.

**Figure 4:**
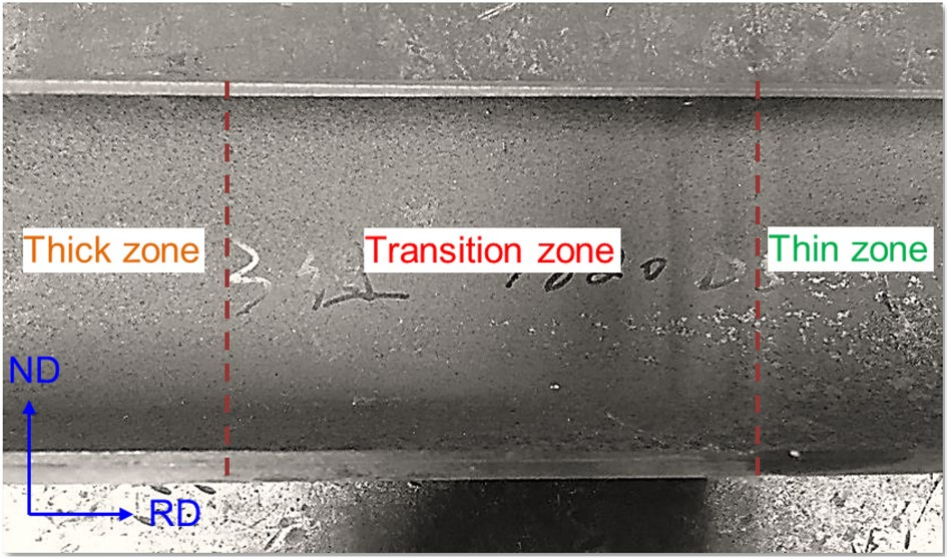
TRT product produced by tailor rolling methods and subsequent forming.

As illustrated in Fig. 4, an actual TRT produced via VGR and sub-forming techniques is presented. The axial thickness variations—aligned with the rolling direction—are discernible, while surface irregularities related to thickness transitions are virtually absent. Notably, the thickness within the transition zone varies linearly, continuously, and smoothly, mitigating the likelihood of abrupt load transitions. By fine-tuning process parameters and coordinating between steps, a variety of TRTs with different dimensions have been fabricated under this geometric framework. Both geometric and form deviations remain within acceptable limits.

#### Characterization method for mechanical property distribution in transition zone

**Figure 5:**
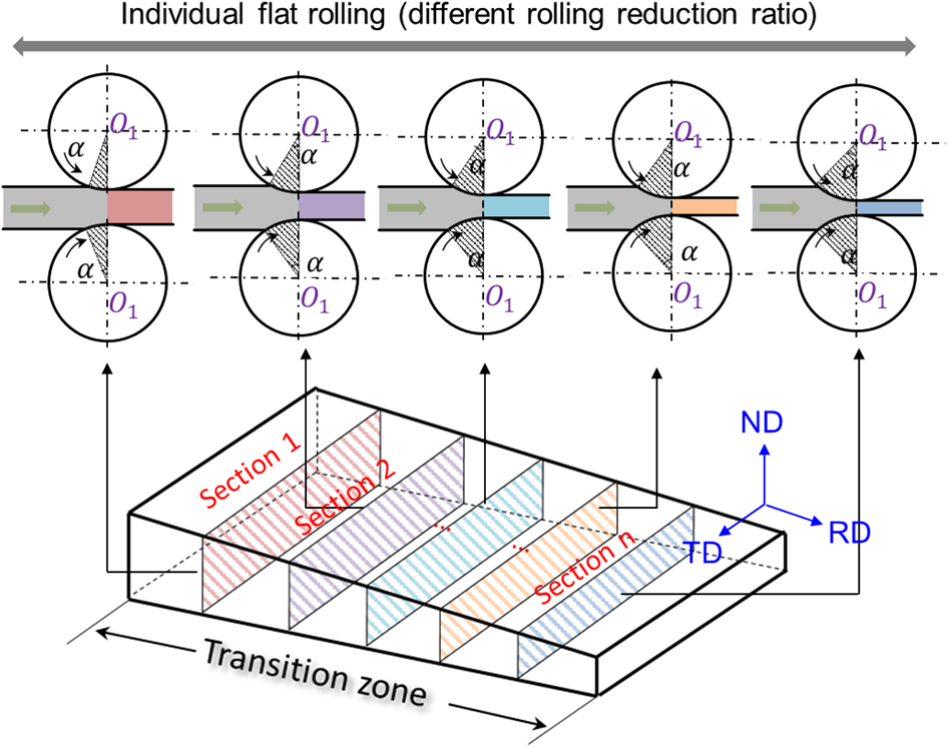
Discrete processing method for identifying mechanical properties of transition zone.

In the VGR process, it is evident that varying degrees of work hardening manifest at disparate positions within the transition zone, attributable to differences in cold rolling reduction ratios, as illustrated in Fig. 5. This results in a pronounced heterogeneity in mechanical properties across the transition zone. Given the continuously fluctuating thickness and mechanical attributes of TRBs, traditional tensile specimens prove inadequate for characterizing stress–strain relations in regions with variable thickness due to the marked non-uniformity in deformation distribution under uniaxial tension. In this context, the FEM provides a suitable discretization framework for capturing the nuanced mechanical behavior within these variable thickness zones. Specifically, complex geometries are discretized into a finite array of elements with rudimentary shapes, thereby transforming the global problem into manageable, elemental computations. Consequently, the transition zone in a TRB can be conceptualized as an assemblage of discrete, equal-thickness plates, each possessing uniform mechanical properties. Mechanical parameters for these individual plates can be derived from uniaxial tensile tests, allowing for the generation of a three-dimensional stress–strain-thickness surface plot. The granularity of this discretization is a determinant of its accuracy. To illustrate, for a TRB with thin and thick zones of 1 mm and 2 mm thickness, respectively, and a longitudinal transition zone length of 100 mm, a total of 11 discrete sections are selected within the transition zones, commencing from 1 mm and incrementing by 0.1 mm intervals. This selection criterion ensures the fidelity of the mechanical property distribution approximations for the transition zone.

**Figure 6:**
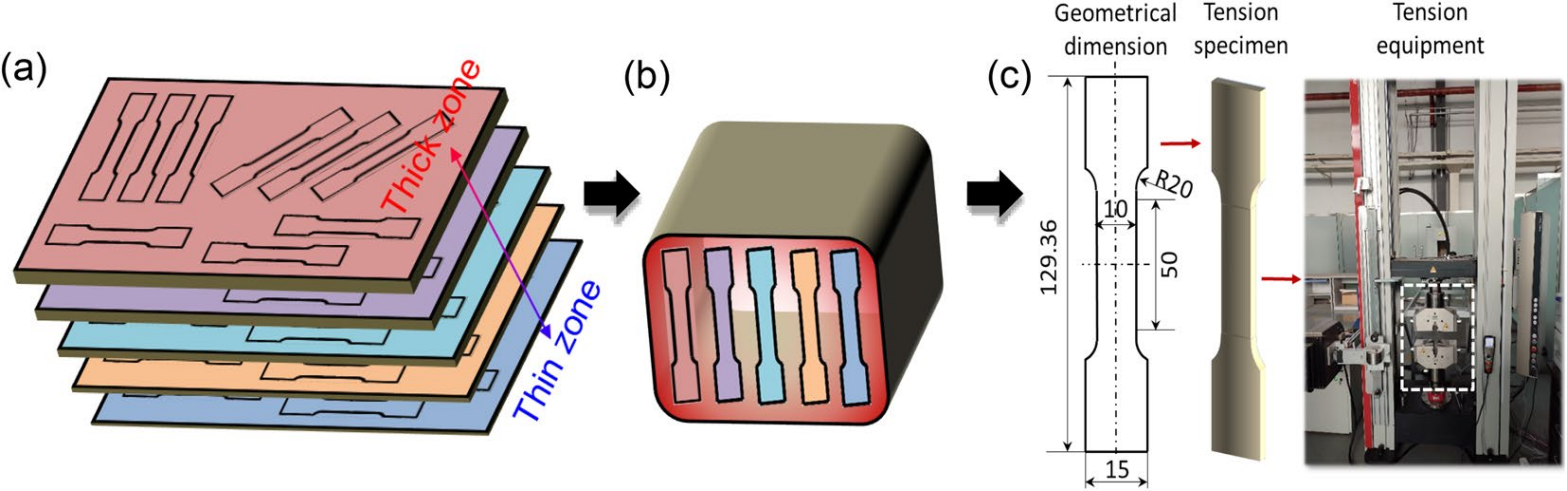
Testing procedure for obtaining mechanical properties of transition zone. (**a**) Sampling location; (**b**) annealing process; (**c**) Uni-axial tension.

As depicted in Fig. 6a, a collection of 11 plates featuring diverse, yet uniform, thicknesses—integral to the transition zone—are fabricated through a series of isolated flat rolling processes. Subsequently, standard tensile specimens are extracted utilizing wire-electrode cutting techniques, with geometric parameters established in compliance with GB/T 288.1—2010 standards. The specific geometric parameters are further elucidated in Fig. 6c, with a minimum of three tensile specimens prepared to ascertain result repeatability. Post VGR, the TRBs are subjected to annealing processes to satisfy diverse performance criteria and to partially alleviate work-hardening, thereby facilitating subsequent deep forming operations, as illustrated in Fig. 6b. Following this treatment, uniaxial tensile experiments are conducted on these specimens employing a Zwick-100 universal testing machine, with a preset tensile velocity of 2 mm/min. The resultant data are then used to construct a curve delineating the relationship between yield strength and thickness.

#### Annealing process

**Figure 7:**
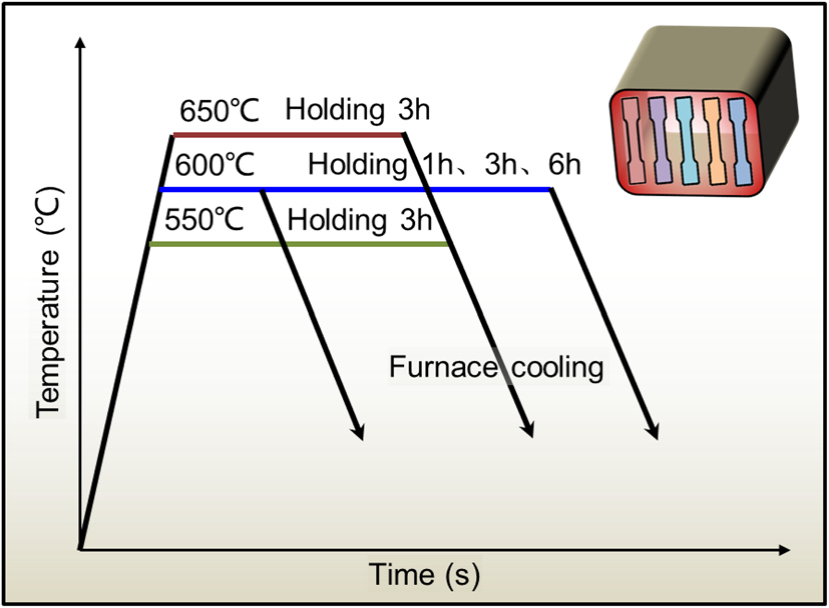
Designed annealing processes for the cold rolled TRB specimens.

The test employs HC340LA steel as the raw material blank, possessing a thickness of 2.2 mm and a chemical composition (by mass fraction, %) as follows: C 0.08, Si 0.21, Mn 0.88, Al 0.047, P ≤ 0.025, S ≤ 0.025, with the remainder being Fe. The annealing procedure for the TRBs diverges from that of traditional, uniform-thickness blanks and presents unique characteristics and challenges: Firstly. Distinct initial mechanical properties are evident in the thin and thick zones of the cold-rolled TRB prior to the annealing process. Secondly. The annealing process must yield a mechanical property distribution in the transition zone that satisfies diverse performance criteria. Utilizing metallography hardness and thermal dilation methodologies (Zhang 2020), the onset temperatures for ferrite-to-austenite transformation (Ac1) and recrystallization temperature (RT) in the experimental TRB with HC340LA were ascertained. Ac1 was identified to be approximately 740 °C, while the RT ranged from 594 to 683 °C, contingent on the degree of reduction. Consequently, based on the established Ac1 and RT values, a series of simulated batch annealing processes were conducted at temperatures ranging from 550 to 650 °C for durations of 60–360 min in a box-type furnace, followed by furnace cooling. These processes are depicted in Fig. 7. The primary objective of investigating these annealing procedures is to align them with industrial production requirements, offering the flexibility for customization without necessitating additional equipment or production lines.

### Mechanical property distribution of tailor structure under annealing process

#### Yield stress distribution

**Figure 8:**
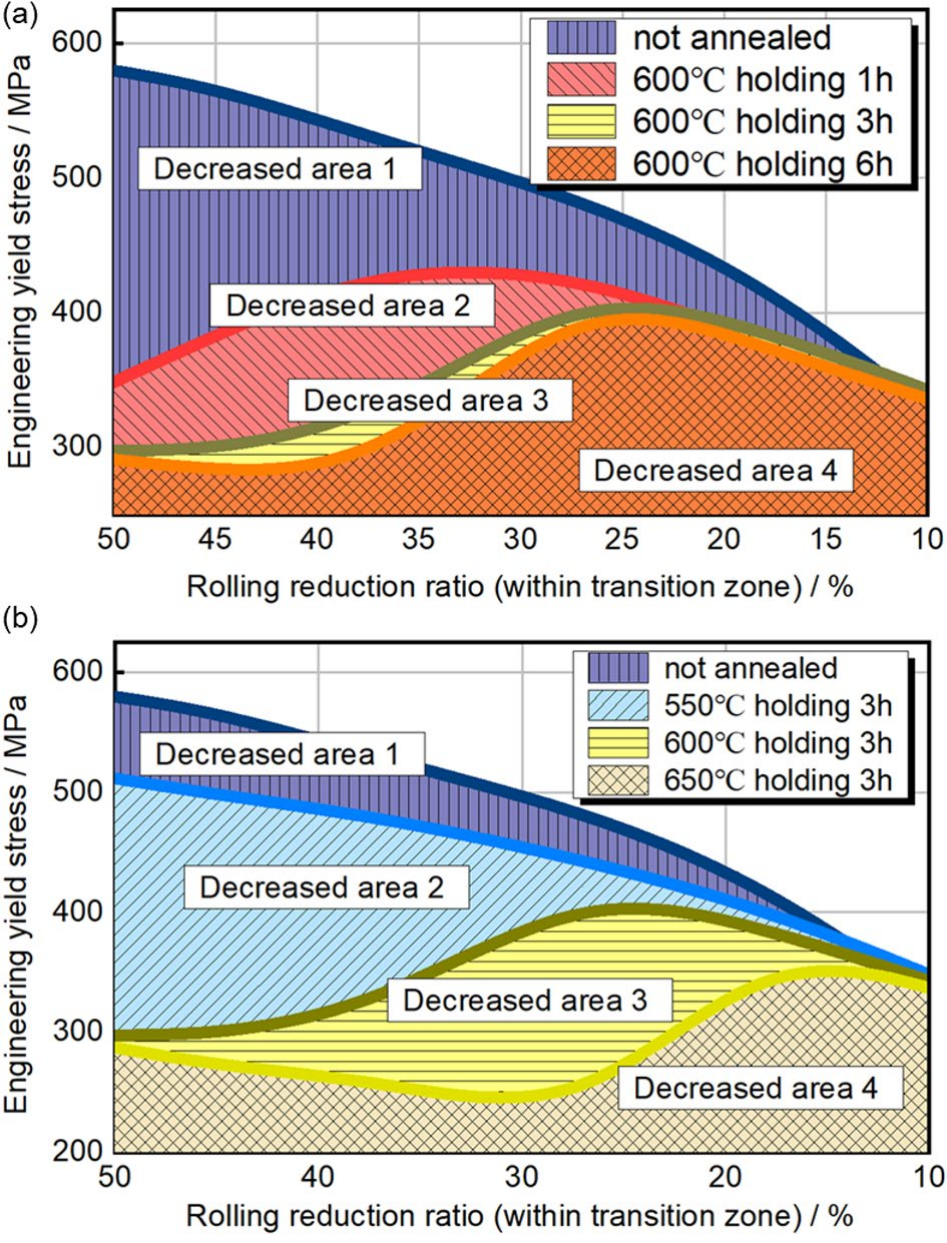
Engineering yield stress distribution of transition zone in TRB after annealing. (**a**) Different annealing holding time; (**b**) different annealing temperature.

The mechanical property distribution of HC340LA TRB post-annealing is presented in Fig. 8. Figure 8a delineates the relationship between annealing time, deformation, and yield strength, while Fig. 8b associates annealing temperature with these parameters. Data suggests incomplete recrystallization with deformation rates from 0 to 50%. Without annealing, mechanical strength decreases exponentially from 580 to 340 MPa as the rolling reduction ratio reduces from 50 to 0%. This indicates enhanced mechanical strength on the thin side of the transition zone due to dislocation interactions. The overall softening degree increases with annealing durations of 1, 3, and 6 h. At 600 °C annealing for 1 h, yield strength peaks at 429 MPa at a 33% rolling reduction ratio. With extended annealing, the peak yield strength decreases and shifts to lower rolling reduction ratios. Complete recrystallization occurs between rolling reduction ratios of 50% and 44% or 39% with longer annealing. Annealing temperature impacts softening more than duration. At 650 °C for 3 h, the peak yield strength is 351 MPa at a 15% rolling reduction ratio, with values below 300 MPa between 50 and 22%. The position of maximum strength shifts toward lower rolling reduction ratios with increased annealing, aligning with the material’s original strength distribution.

#### Equivalent strength distribution

**Figure 9:**
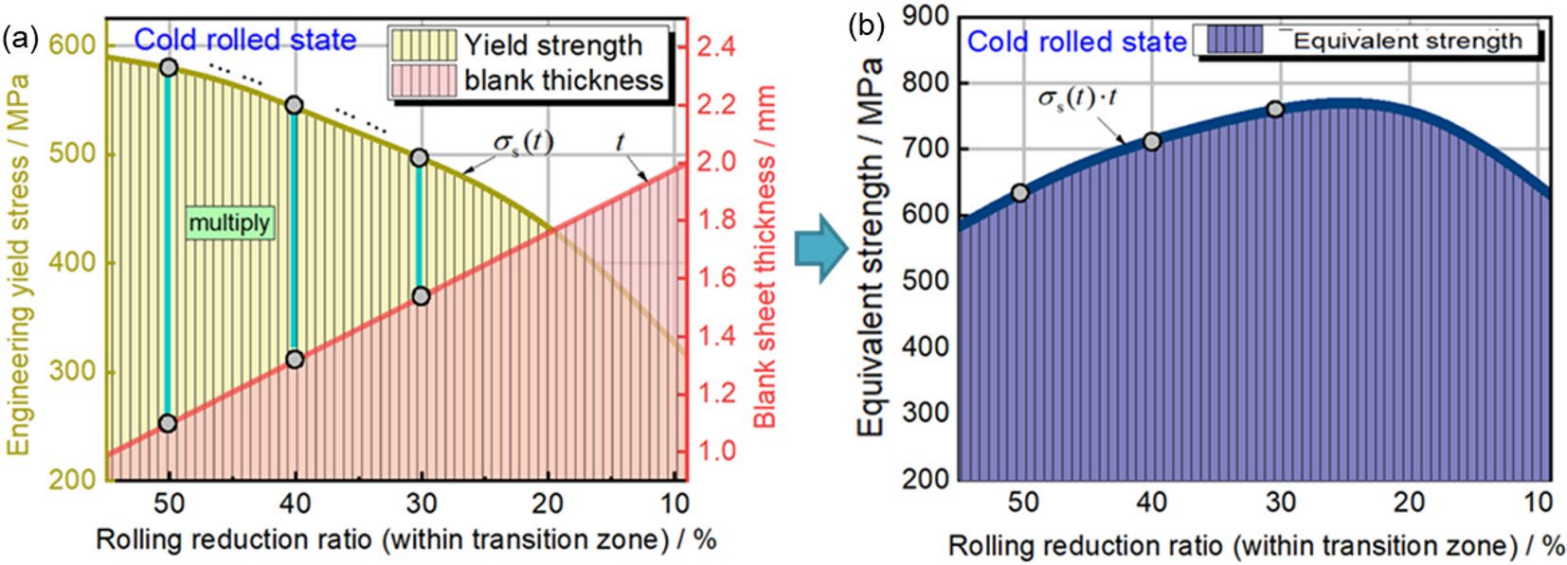
Description of equivalent strength distribution of transition zone in TRB. (**a**) Distribution of engineering yield stress and thickness; (**b**) equivalent strength distribution.

The unique tailor-made components are achieved through subsequent forming of TRB, leading to an uneven thickness distribution. As a result, determining the potential occurrence of instability cannot solely rely on the yield strength distribution under real-world service conditions. As depicted in Fig. 9, a fresh parameter is introduced to characterize the load-bearing capability at each thickness location in the transition zone. In this study, we propose the term “equivalent strength”, obtained by multiplying the yield strength with the corresponding thickness, to represent the fundamental load-bearing potential. It’s important to highlight that this distribution of equivalent strength must differ from that of yield strength. It is worth further investigating whether the point of structural instability corresponds to the lowest equivalent strength position across various external loading scenarios. Additionally, attention must be given to the ensuing deformation and mechanical response of the structure.

**Figure 10:**
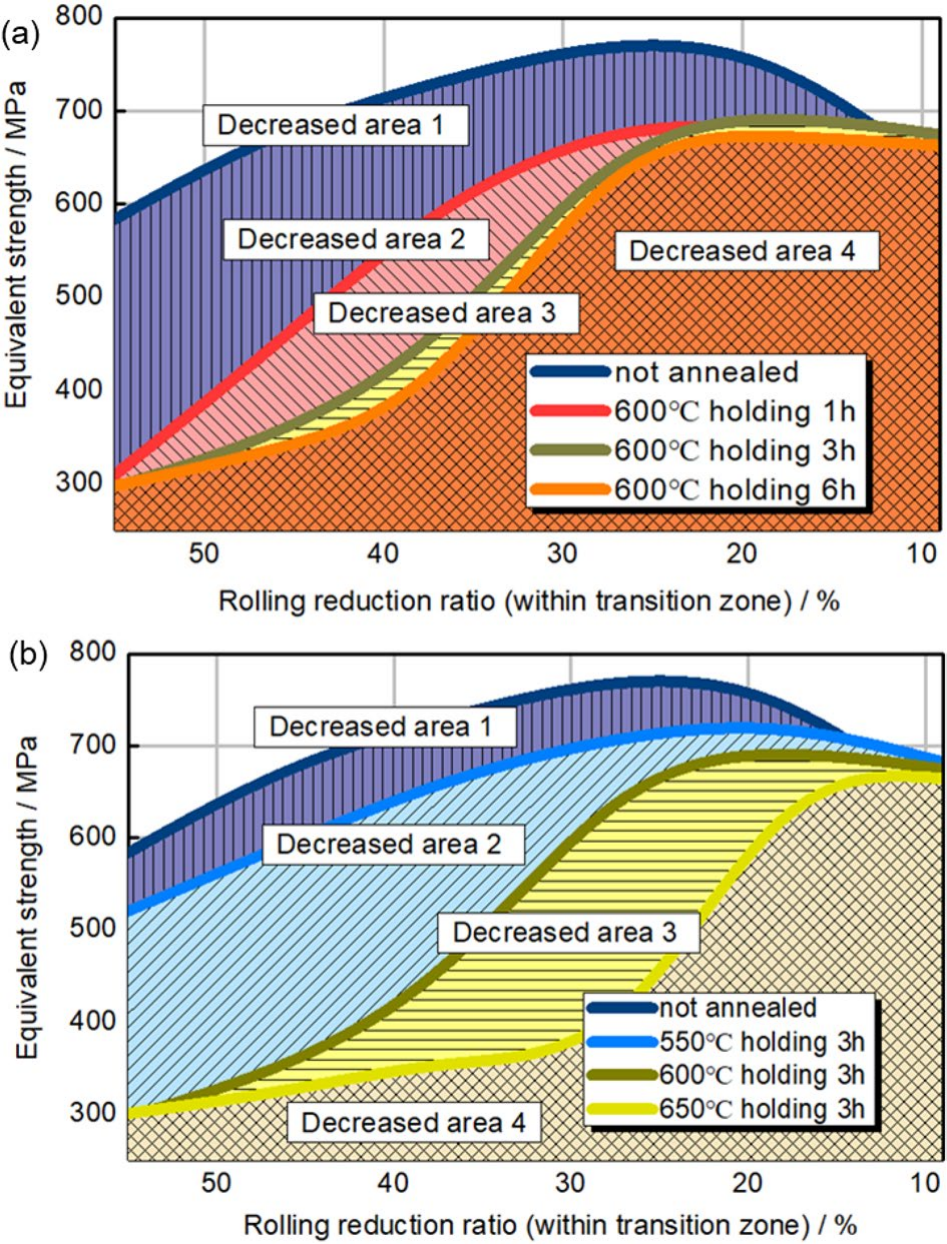
Equivalent strength distribution of transition zone in TRB after annealing. (**a**) Different annealing holding time; (**b**) different annealing temperature.

As depicted in Fig. 10, the distribution of equivalent strength within the TRB’s transition zone following incomplete recrystallization annealing is illustrated. Beyond the cold-rolled state, the equivalent strength predominantly exhibits a consistent upward trend as the rolling reduction ratio diminishes. Due to the exponential nature of yield strength distribution, the equivalent strength distribution in the cold-rolled state takes on the form of a Gaussian curve, with its zenith occurring at the 24% rolling reduction ratio mark. As annealing temperature or duration increases, the equivalent strength distribution curve transitions from a Gaussian profile to a Boltzmann-type curve. Notably, the minimum equivalent strength consistently emerges at the position characterized by the highest rolling reduction ratio, whether in the context of incomplete or complete annealing processes. In contrast to Fig. 8, the location of minimum yield strength shifts from high rolling reduction ratio positions to those with lower rolling reduction ratios as annealing temperature or time escalates, marking a substantial contrast with Fig. 10. This underscores the paramount influence of the thickness factor on load-bearing capacity, surpassing that of mechanical strength. Consequently, when designing components with variable thickness distributions, the foremost consideration should remain the thickness distribution factor.

### Uni-direction tensile of TRB

#### Validation of the FE modeling

**Figure 11:**
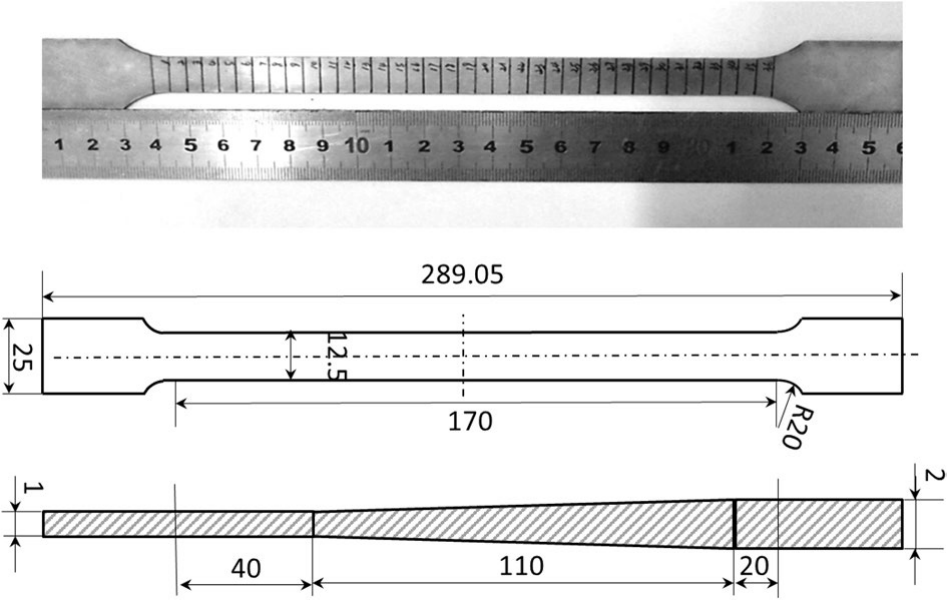
Geometric size of uniaxial tensile sample for TRBs.

**Figure 12:**
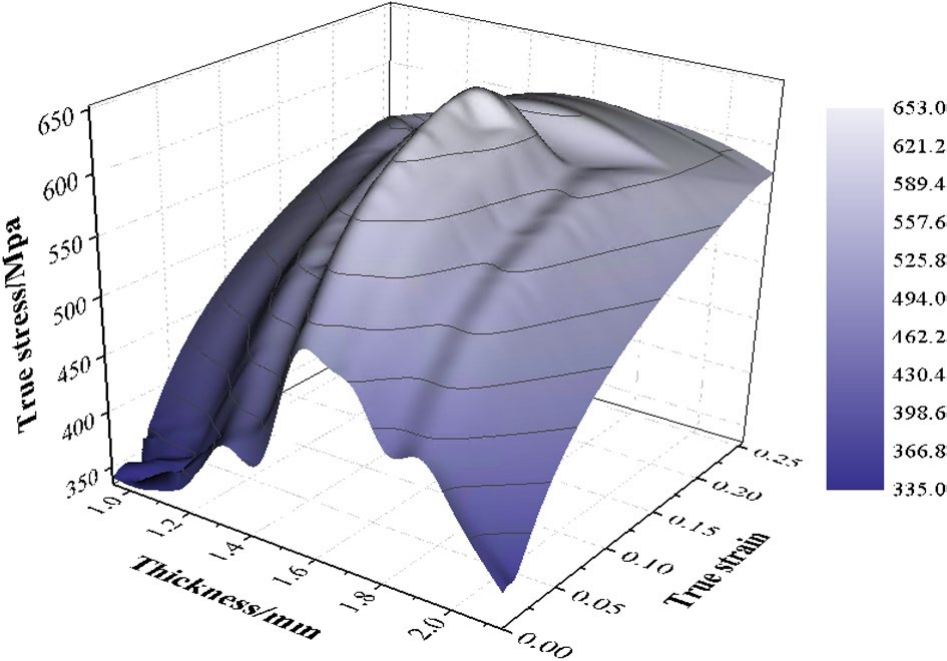
Mechanical property distribution of transition zone in TRB after annealing at 590 °C for 5 h.

In pursuit of a more comprehensive evaluation of fundamental mechanical attributes and FEM accuracy, an innovative elongated tensile specimen is conceived, encompassing both the transition zone and a portion of the uniform thickness region. This specimen is fashioned through the wire-electrode cutting technique, as depicted in Fig. 11, with dimensions conforming to GB/t 288.1-2010 standards. To align with the mechanical requirements of engineering industries, a 590 °C annealing process over 5 h is implemented for the TRB tensile specimen. The earlier-discussed discretization concept remains applicable, facilitating the characterization of the mechanical properties of the variable thickness zone. Upon data processing, the mechanical property distribution of the transition zone in TRB (HC340LA) is illustrated in Fig. 12. For the TRB tensile sample’s surface, a 10% hydrochloric acid solution is employed to eliminate heat treatment-induced iron oxide accumulation.

The dynamic explicit non-linear FEM for simulating the tensile process of TRB is established using ABQUS/CAE 2021 software. The geometric configurations in simulation align with the experimental setup. Essential mechanical properties of TRB include: density *ρ* = 7850 kg/m^3^, elastic modulus *E* = 210 GPa, and Poisson’s ratio *v* = 0.33. The material model considers the relationship between yield stress and plastic strain with thickness to define plastic properties of TRB. The simulation employs a clamping end kinematic coupling, with one end having six degrees of freedom constrained. A tensile displacement of 25 mm is applied to the central reference point of the other clamping end. The analysis step spans 25 s. Four-node shell elements S4R with 9 integration points through the thickness represent the TRBs. The element size is set as 1 mm for the parallel segment and 2 mm for clamping ends, ensuring a balance between efficiency and accuracy. Analytical fields are used to assign thickness and mechanical property distributions in the transition zone, ensuring the model’s accuracy. The comparison of kinetic energy to internal energy highlights the negligible influence of inertial forces, rendering the tensile process quasi-static.

Tensile tests on the TRB were performed by first cutting specimens from the material produced via VGR, which were then annealed at 590 °C for 5 h and subjected to uniaxial tensile testing to obtain actual performance data. To capture the actual mechanical property characteristics of the thickness transition zone, the approach involved selecting 11 equidistant thickness positions along the length of the transition zone. Sheets of uniform thickness were produced for these positions through traditional rolling methods, followed by annealing and cutting. Uniaxial tensile tests were then conducted to obtain stress–strain curves for each position. For areas outside these 11 positions within the transition zone, stress–strain curves were interpolated. The finite element simulation of the tensile behavior for the TRB is divided into three key steps: setting the thickness distribution, assigning mechanical properties, and linking these properties with thickness variations. The transition zone is discretized identically into 11 segments, each 10 mm long, with 0.1 mm incremental changes in thickness, assigned through analytical field method. Linear interpolation is used for areas between these segments to accurately reflect the material’s thickness variations. The properties of the 11 discrete thickness positions, such as stress–strain data, are derived using the discretization method previously detailed in Figs. 5 and 6. These properties are then entered into the mechanical properties module of the finite element program to ensure accurate simulation results. An intermediate variable was introduced in the simulation to create a direct link between the thickness variations and their corresponding mechanical properties, ensuring each thickness level accurately reflects its specific material characteristics. For thickness positions other than the 11 specifically defined segments, properties are assigned using interpolation to approximate the variations.

**Figure 13:**
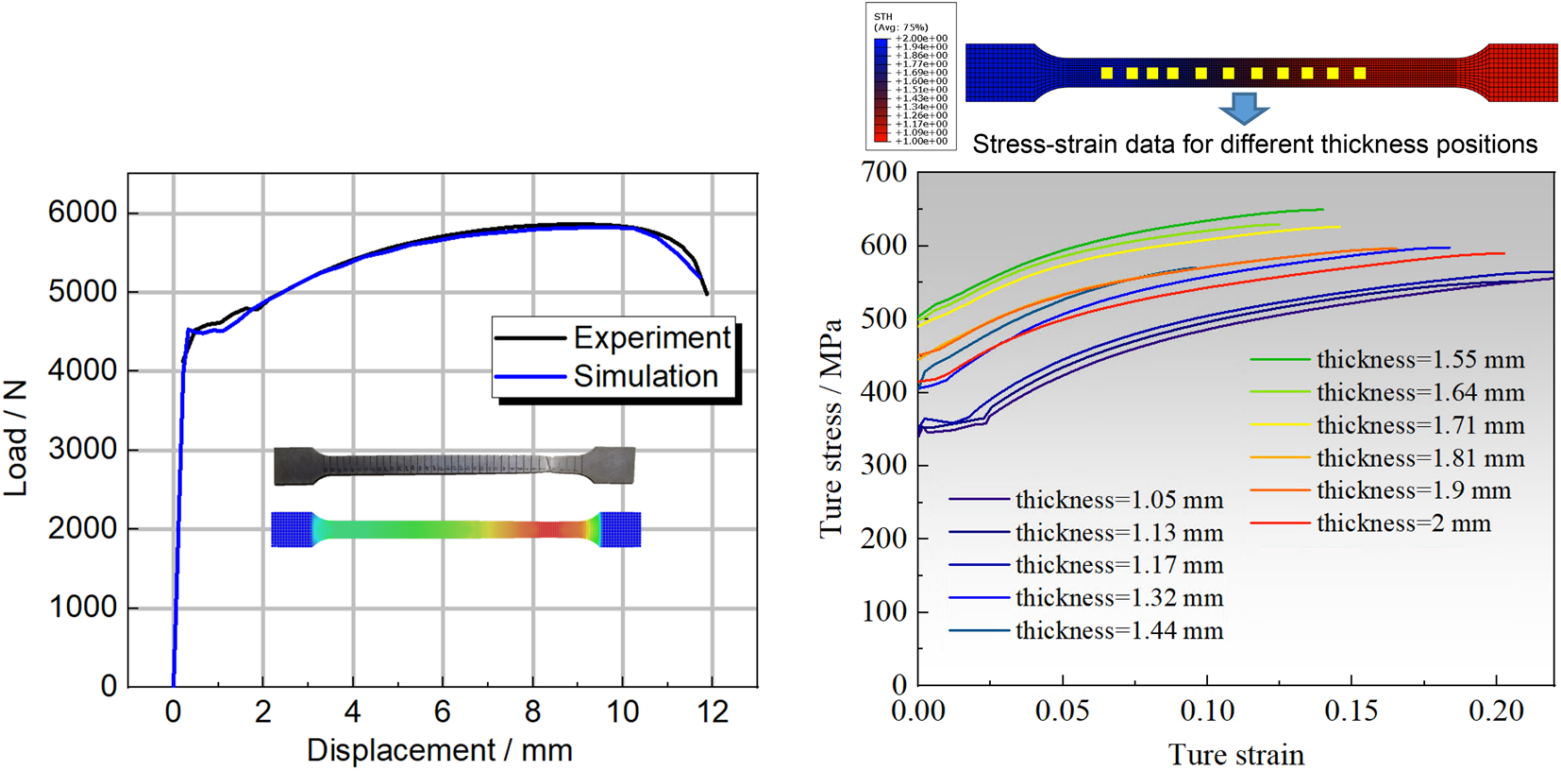
Load–displacement curve of TRB (HC340LA) by experiment and simulation, including stress–strain curves for different thickness positions.

This methodology, applied to the actual TRB specimen and its finite element model as shown in Fig. 13, accurately reflects stress–strain data across varying thicknesses, thereby providing a comprehensive representation of the TRB’s overall tensile performance and deformation characteristics. The load–displacement curve from both experimental and simulated TRB tensile processes is also displayed in Fig. 13. The excellent alignment, especially during plastic deformation, underscores the accuracy and reliability of the mechanical property data obtained from prior tensile tests and the finite element model for TRB’s tensile process based on this data. Additionally, this foundational approach was consistently applied to model the thickness and property distributions in subsequent design iterations.

**Figure 14:**
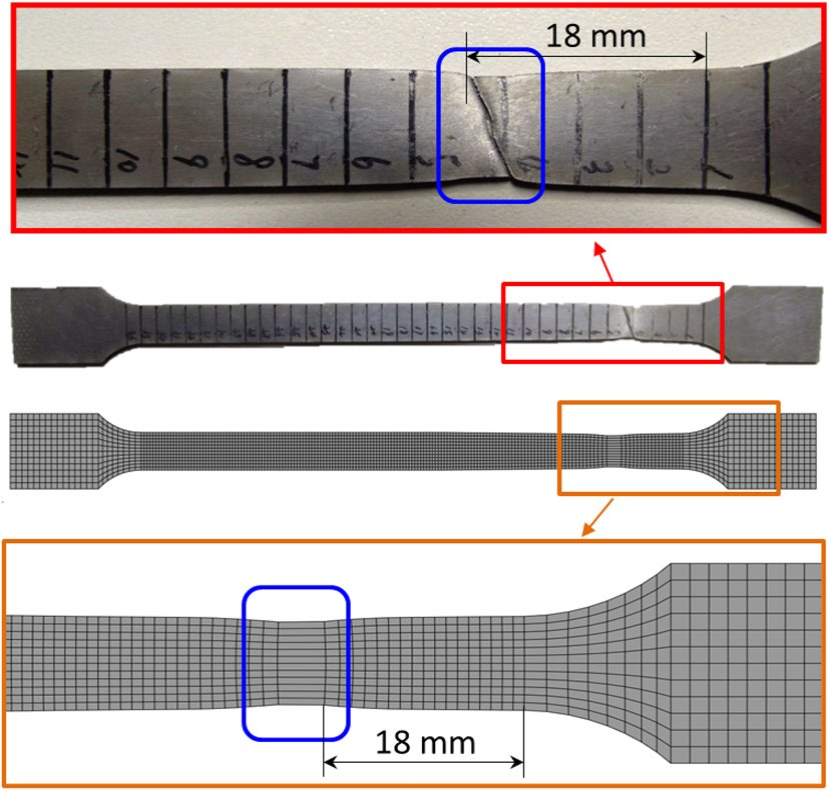
Instability position of tensile TRB (HC340LA) by experiment and simulation.

As depicted in Fig. 14, a comparison between the fracture location of the actual TRB tensile specimen and the instability position predicted by the FEM for TRB’s tensile process is evident. Notably, the FEM demonstrates a commendable capability to accurately anticipate instability and fracture occurrence. Furthermore, the FEM facilitates an insightful understanding of the stress and strain response throughout the TRB tensile process, thereby furnishing essential insights for subsequent tailor product design endeavors. The analysis underscores that the instability within TRB emerges predominantly in the thin zone due to its relatively weaker load-bearing capacity. While the established FEM does not incorporate fracture models or grain orientations, it efficiently predicts the instability location and mechanical response. Consequently, this FEM serves as a valuable tool for forecasting macroscopic mechanical responses and deformation behaviors, despite its lack of representation of crack morphology and propagation direction.

#### Designed mechanical property distributions

**Figure 15:**
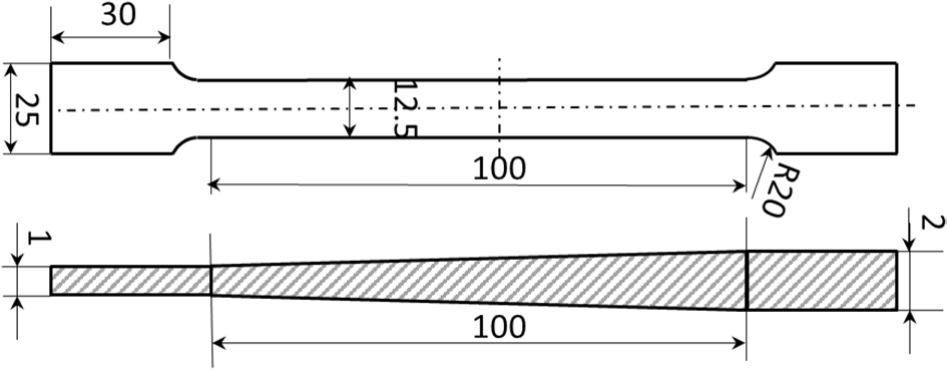
Geometric size of uniaxial tensile sample for TRB by FEM.

**Figure 16:**
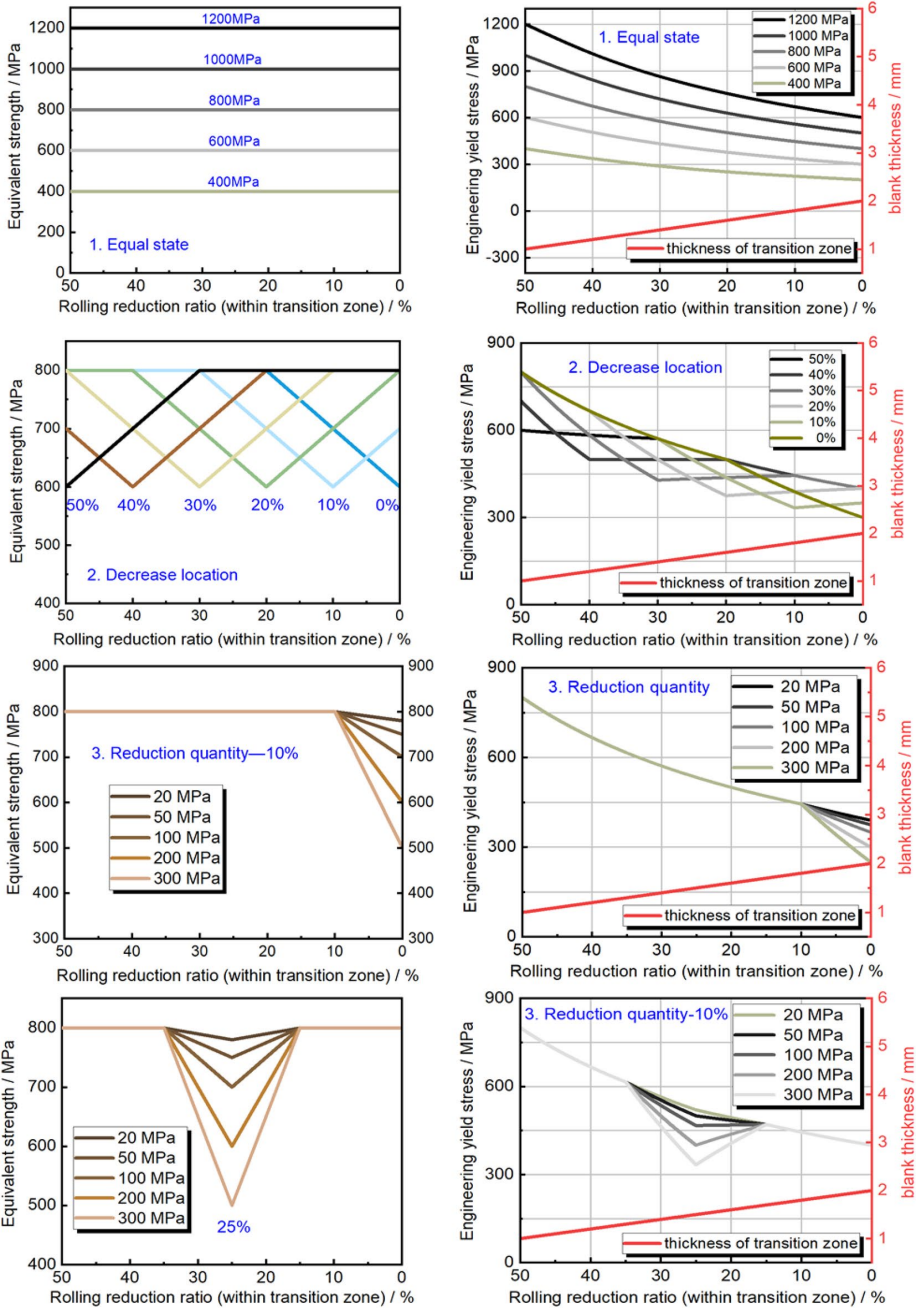
Geometric size of uniaxial tensile sample for uniform thickness blanks (UB).

**Figure 17:**
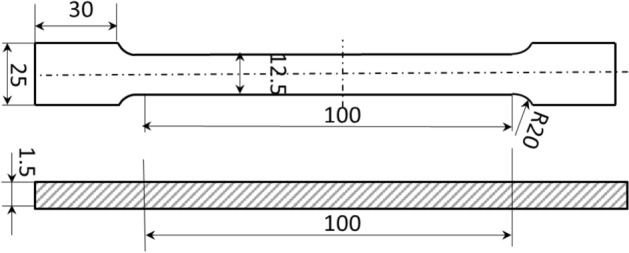
Designed engineering yield stress distribution and equivalent strength distribution of transition zone in TRBs and TRTs.

It is evident that the thin zone exhibits the lowest equivalent strength, suggesting a susceptibility to instability and fracture from this standpoint. However, if the weakest equivalent strength is not situated within the thin zone due to diverse equivalent strength distributions influenced by varying heat treatment methods, the possibility of instability arising elsewhere becomes substantial. To probe the ramifications of distinct equivalent strength distributions on structural instability locales and fundamental mechanical responses, an array of tailored mechanical property distributions is imposed upon the tensile samples, encompassing both continuous variable thickness and equal thickness conditions. As illustrated in Figs. 15, 16 and 17, two variants of tensile samples are presented. To ensure comparability, the dimensions of the parallel segments in both types remain consistent, preserving equivalent volumes. Within TRB tensile samples, the entire thickness transition zone occupies the parallel segment, obviating the presence of discrete thin and thick zones with uniform thickness. Refer to Figs. 15 and 17 for specific geometric dimensions. Notably, the devised mechanical property distributions are meticulously engineered and imparted onto the tensile samples. When assigning designed mechanical property distributions to tensile specimens in the finite element model, the Ludwik equation is employed to characterize the stress–strain relationships at various thickness positions. The material strengthening coefficient and strain hardening exponent are set at 630 and 0.66, respectively. These values are determined by fitting formulas to stress–strain curves from different thicknesses and then calculating their arithmetic mean. Consequently, in the design of mechanical property distributions, only the yield strength and flow stress are varied, allowing for a focused examination of the primary factors of interest. While achieving these designated mechanical property distributions through practical means is challenging, emerging techniques like local induction heating and selective laser heat treatment hold promise for realizing such objectives. The FEM is employed herein to scrutinize the influence of equivalent strength distributions on structural instability locations and core mechanical responses. This approach primarily aims to mitigate potential responses stemming from local geometric imperfections or microstructural heterogeneity to deformation and mechanical properties.

**Figure 18:**
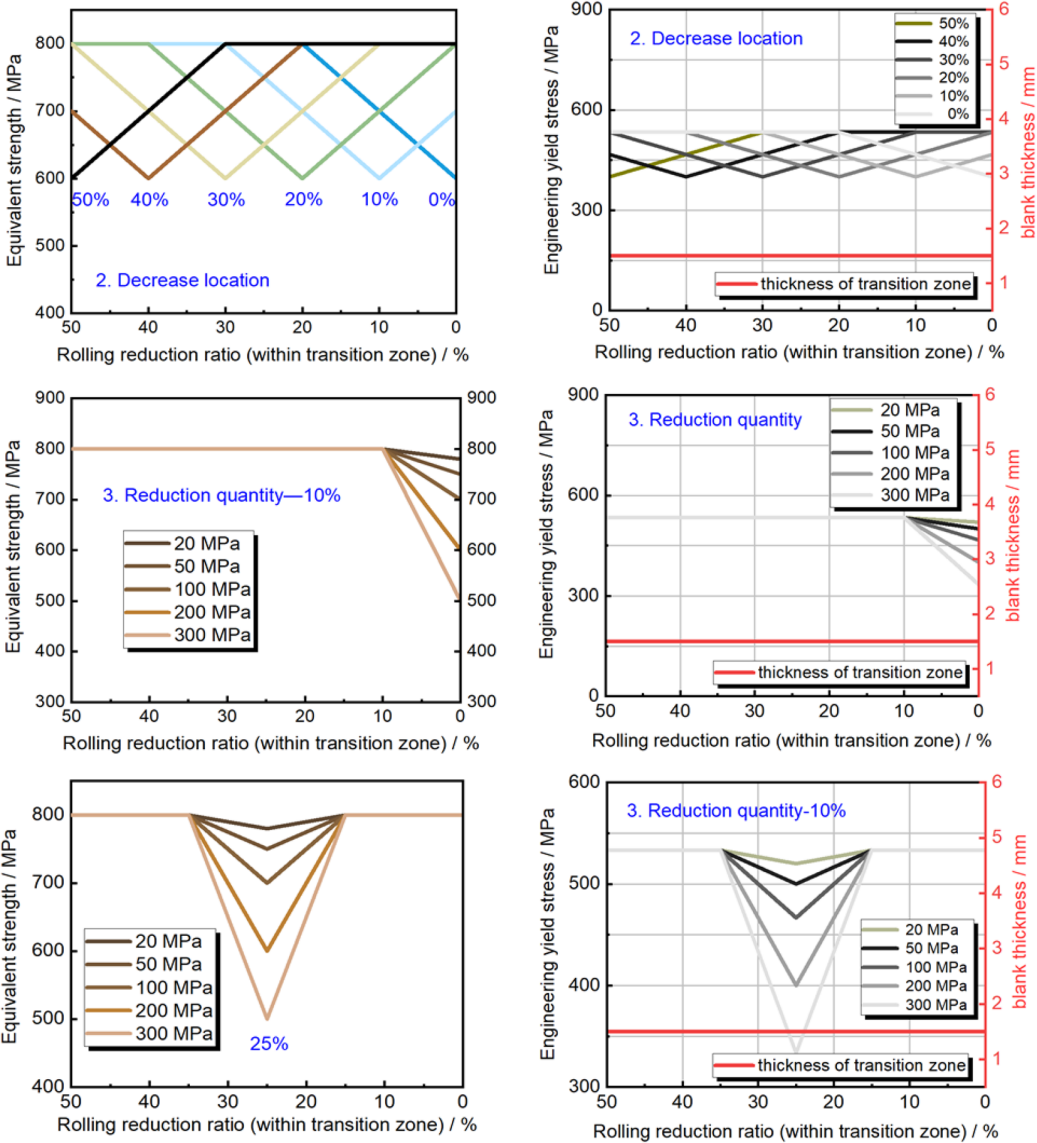
Designed engineering yield stress distribution and equivalent strength distribution in uniform thickness blanks (UB).

The thickness distribution within the parallel segment of the tensile TRB linearly transitions from 1 to 2 mm across a length of 100 mm. This distribution remains consistent across various equivalent strength profiles. Figure 16 illustrates the application of four distinct equivalent strength distributions, including the actual in-complete annealing property mentioned earlier (as illustrated in Fig. 10), onto the uniaxial tensile TRB samples. Among these, the aforementioned actual equivalent strength distribution type is used, while the other four designed types include equal strength, location-based reduction, end-based reduction, and middle-based reduction. The left side of Fig. 18 showcases the equivalent strength distributions, while the right side depicts the yield strength and thickness distributions along the longitudinal transition zone. For the equivalent strength distributions, a range of equivalent strength levels spanning from 400 to 1200 MPa is implemented to investigate the influence of thickness distribution as a single variable. To explore the impact of localized weaker strength positions, varying locations with a 200 MPa reduction in equivalent strength, ranging from 0 to 50% rolling reduction ratios, are considered. The other two types of equivalent strength distributions are designed to examine how the extent of strength reduction within local positions, spanning from 20 to 300 MPa, affects structural instability locations and fundamental mechanical responses. By employing a series of mechanical property distribution variations, a comprehensive grasp of dual distributions’ effects on mechanical behaviors is obtained. In terms of implementation methods, the recovery annealing method is suitable for realizing the equivalent strength distribution type, while a combination of recovery annealing and local heat treatment technology can be employed for the other types. Conversely, three equivalent strength distribution types, including location-based reduction, end-based reduction, and middle-based reduction (as depicted in Fig. 18), identical to TRB samples, are applied to uniaxial tensile samples with equal thickness (UB) to explore the influence of thickness distribution under different mechanical property distributions. These samples can be produced using local heat treatment technology alone, which is a simpler realization process compared to the dual distribution TRB samples.

#### Effect of mechanical property distributions on plastic instability of TRB

**Figure 19:**
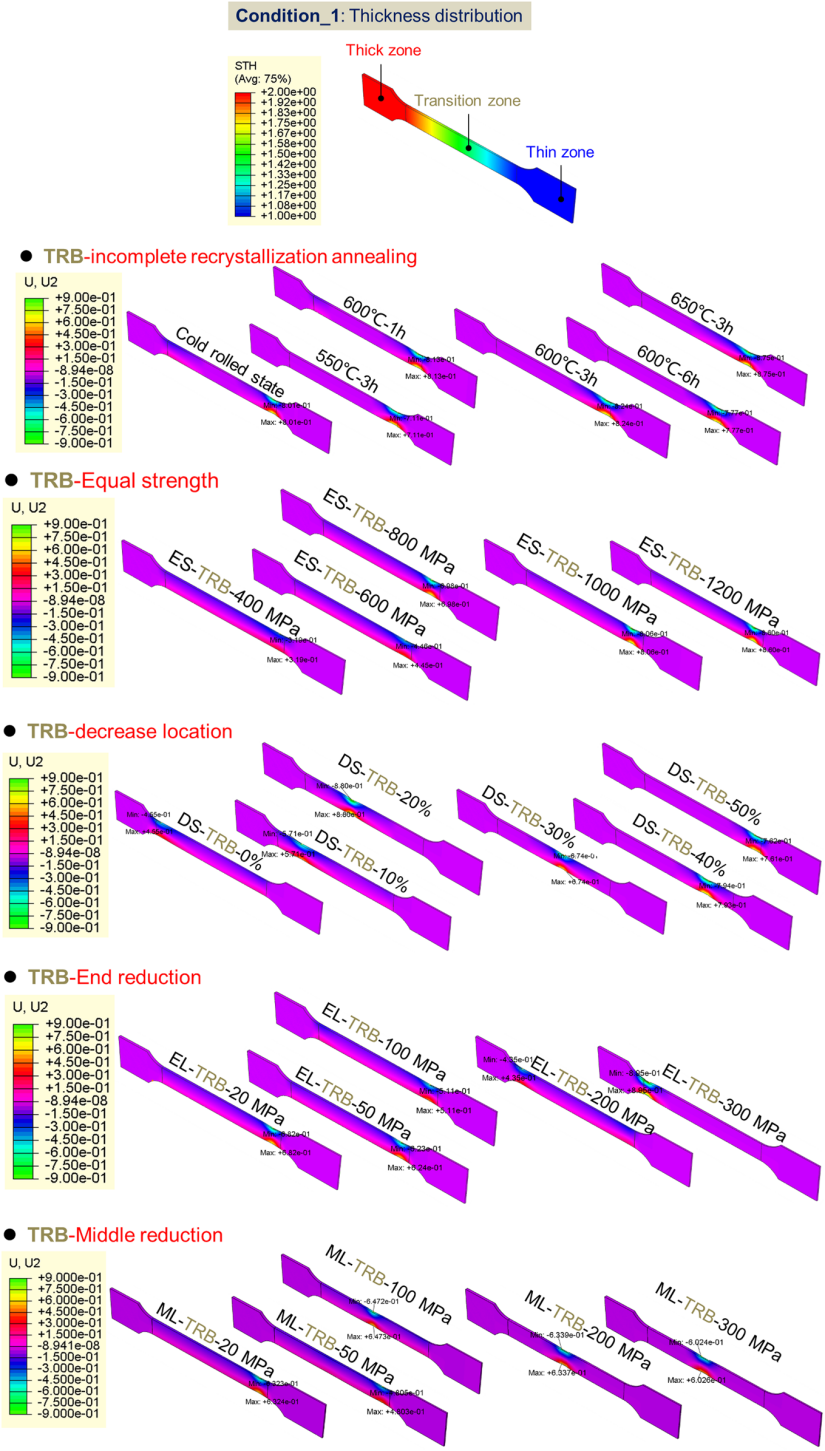
Wide neck shrinkage of TRB under designed mechanical property distributions during tensile process.

**Figure 20:**
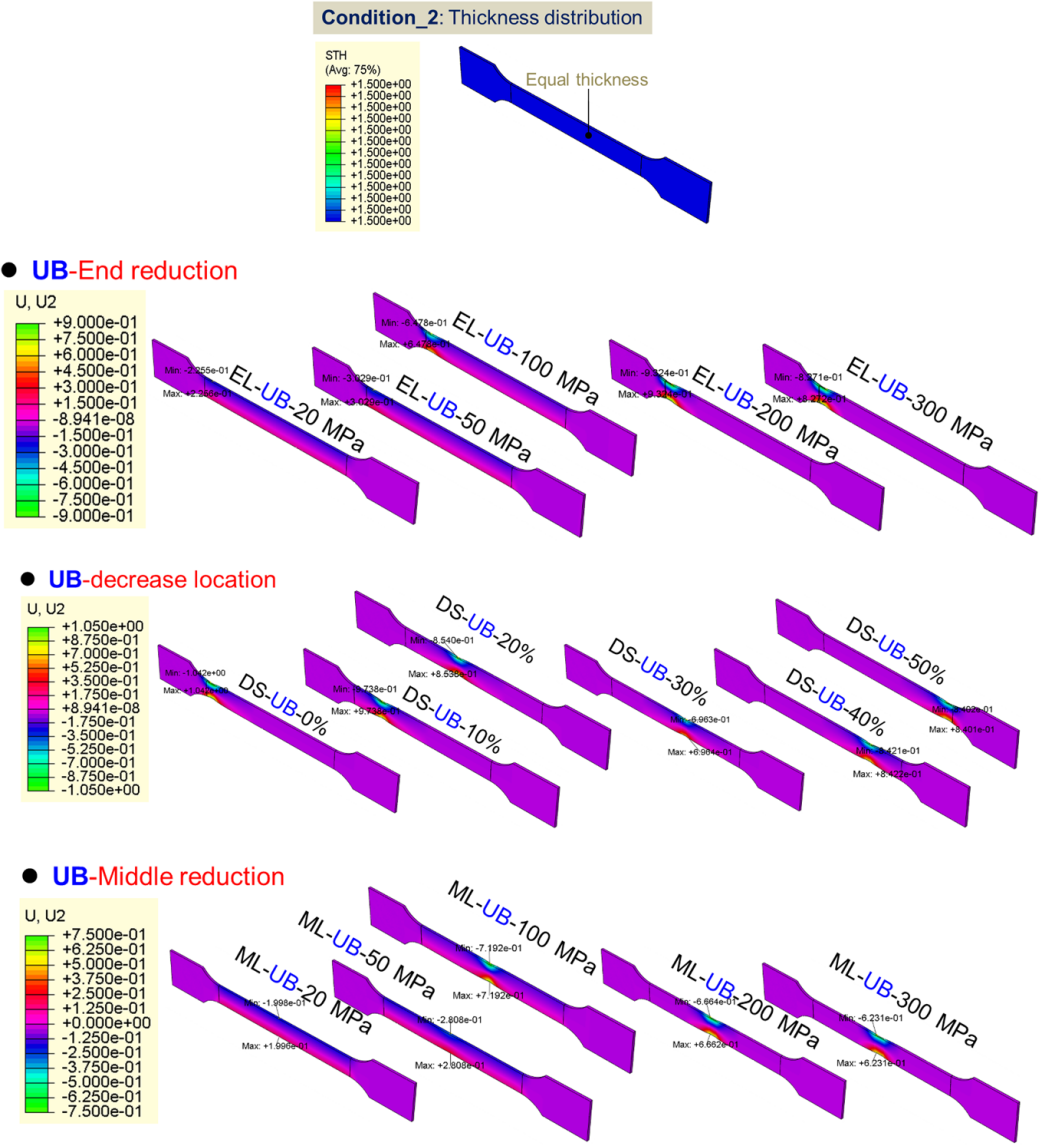
Wide neck shrinkage of UB under designed mechanical property distributions during tensile process.

Research has delved into the plastic instability and mechanical responses of TRB and TRT structures under various loadings, with comparisons to equal thickness structures. This study meticulously evaluates the interplay of thickness and mechanical property distributions, focusing on equivalent strength distribution impacts on deformation patterns (as depicted in Figs. 19, 20). Key insights include: HC340LA steel-based TRBs display intricate mechanical property distributions. Without annealing, strength sharply drops from 580 to 340 MPa as the reduction ratio shifts from 50 to 0%. Enhanced annealing initially raises, then reduces mechanical strength along the transition zone. Instability predominantly appears in the thinnest zone, with heat treatment not effectively countering this deformation. Equivalent strength distributions (400 MPa to 1200 MPa) in TRBs, TRTs, UBs, and UTs reveal instability primarily in the thinnest zone due to its reduced carrying capacity. For axial loading, thickness plays a pivotal role in determining this capacity. Under tensile conditions, TRBs and UBs show instability at locations with reduced strength. TRB’s metal flow capacity can be optimized by balancing strength disparities, while UB structures face compromised deformation coordination. TRTs and UTs exhibit slight variations in instability patterns due to factors like end friction and bending moment dynamics. Fluctuations in equivalent strength influence instability positions. In TRBs, instability shifts between thin, thick, or middle zones based on the extent of strength reduction. In TRTs, coordinated deformation and overlapping buckling elements lead to simultaneous buckling in multiple zones. TRB samples with tailored properties exhibit pronounced non-uniform deformation from the onset, failing to utilize their full carrying capacity. The position with the lowest ultimate equivalent strength is prone to plastic instability. For tailored TRTs, bending moment variations across thicknesses are crucial for accurate instability prediction. This research offers two design guidelines: achieving consistent equivalent strength across thicknesses can improve TRBs’ elongation and performance. Strategically controlling ultimate equivalent stress or bending moment in specific thickness areas can bolster safety in tailored structures.

**Figure 21:**
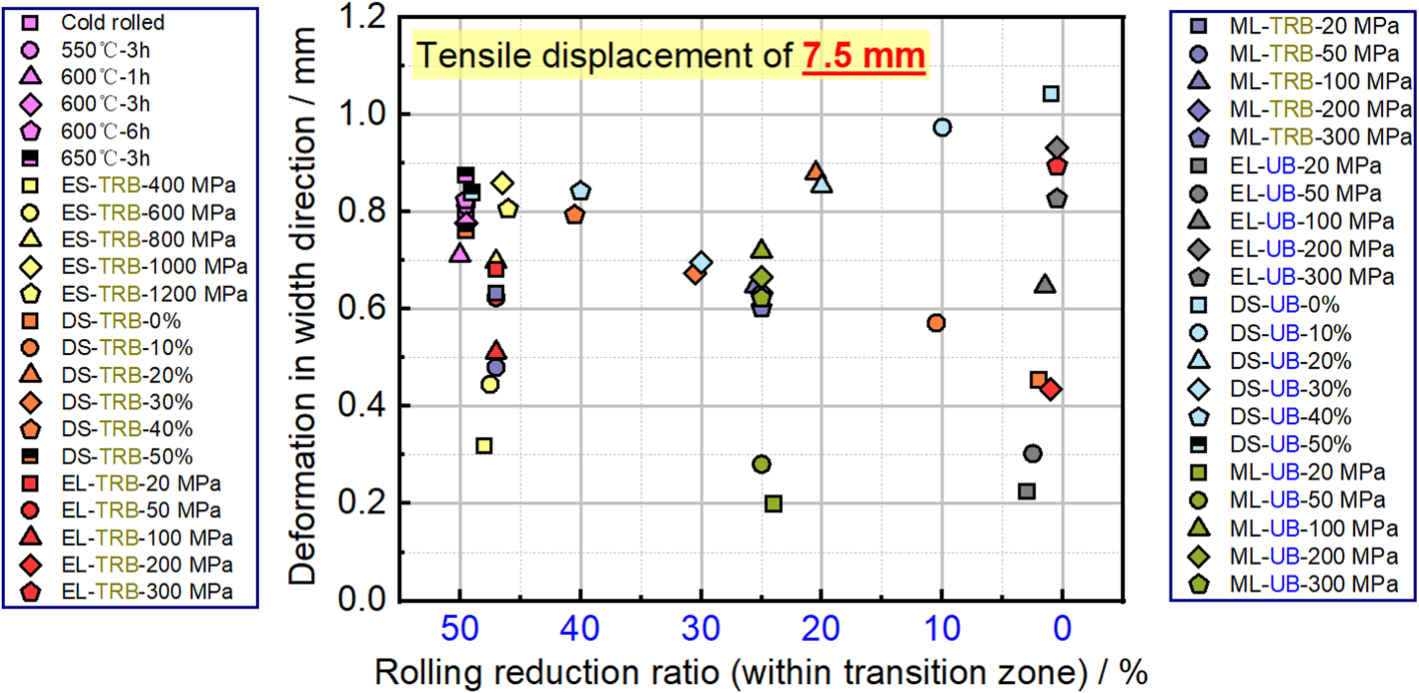
The maximum wide deformations and corresponding locations of tensile samples for TRBs and UBs.

Figure 21 displays the maximum deformations in the width direction for tensile samples. It can be inferred that samples with smaller deformations in width are more resistant to plastic instability. With this in mind, an equal strength distribution is preferable for TRB to enhance its resistance to such instability. Lowering the strength in the thicker areas can lead to better uniform elongation. While an equal strength distribution is beneficial for resisting plastic instability in UB, it makes it difficult to predict where this instability will occur. By using the selective tailor heat treatment technique, we can adjust TRB’s property distribution to better control instability locations, enhancing overall stability. This tailored approach provides a more controlled distribution of force and strain.

**Figure 22:**
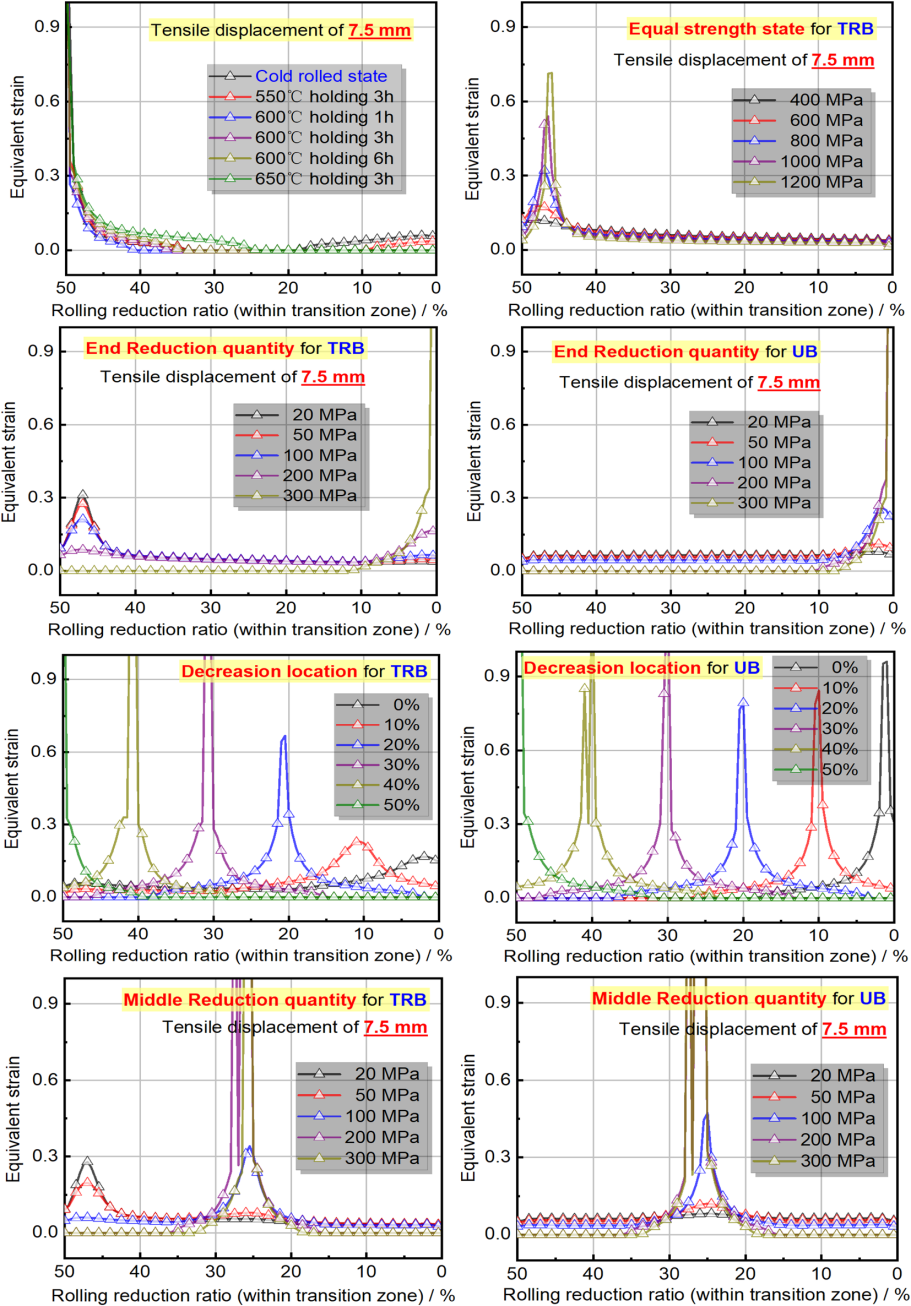
Equivalent strain distributions of TRB and UB with designed mechanical property distributions.

The equivalent strain distribution directly reflects plastic deformation at various points. Figure 22 shows these distributions for TRB and UB under different strength distributions, mirroring widthwise deformation. For TRB under incomplete recrystallization annealing, plastic deformation is evident in both thin and thick zones at temperatures below 550 °C, but is prominent in the thin zone above 600 °C. Below 550 °C, the thin and thick zones have lower strengths, making them more susceptible to deformation. Above this temperature, the thin zone’s strength is significantly lower, leading to pronounced deformation, especially at its thinnest. Notably, the strain in the thinnest zone exceeds 1, indicating significant instability. TRB with equal strength shows higher strains near the thinnest zone, decreasing with reduced rolling ratio. This distribution is more uniform than incomplete recrystallization annealing. The study also explored the effect of local strength reduction. Results show a threshold for instability in TRB’s thin or thick zone between a 100 and 200 MPa reduction. Beyond 200 MPa, deformation shifts to the thick zone, while below 100 MPa, it remains in the thin zone. This highlights the role of thickness in deformation resistance, confirmed by comparing with UB results. TRB under middle strength reduction shows a similar trend, with an instability threshold between 50 and 100 MPa due to the thinner middle zone. For UB, the central zone is in the middle due to equal thickness distribution. However, with over 200 MPa reduction, other areas remain elastically deformed. This indicates TRB’s thicker zone has stronger deformation resistance than UB’s under similar conditions. The maximum strain is in the central instability zone, decreasing as strength reduction shifts from thin to thick. Unlike TRB, UB doesn’t show this pattern, emphasizing the greater impact of thickness over yield strength. Moving strength reduction from thin to thick also improves TRB’s deformation compatibility. The best method to enhance TRB’s deformation capacity involves equal strength distribution and slight strength reduction with increasing thickness. Further research is needed on the exact reduction and layout.

#### Discussion for the uneven deformation mechanism of TRB

**Figure 23:**
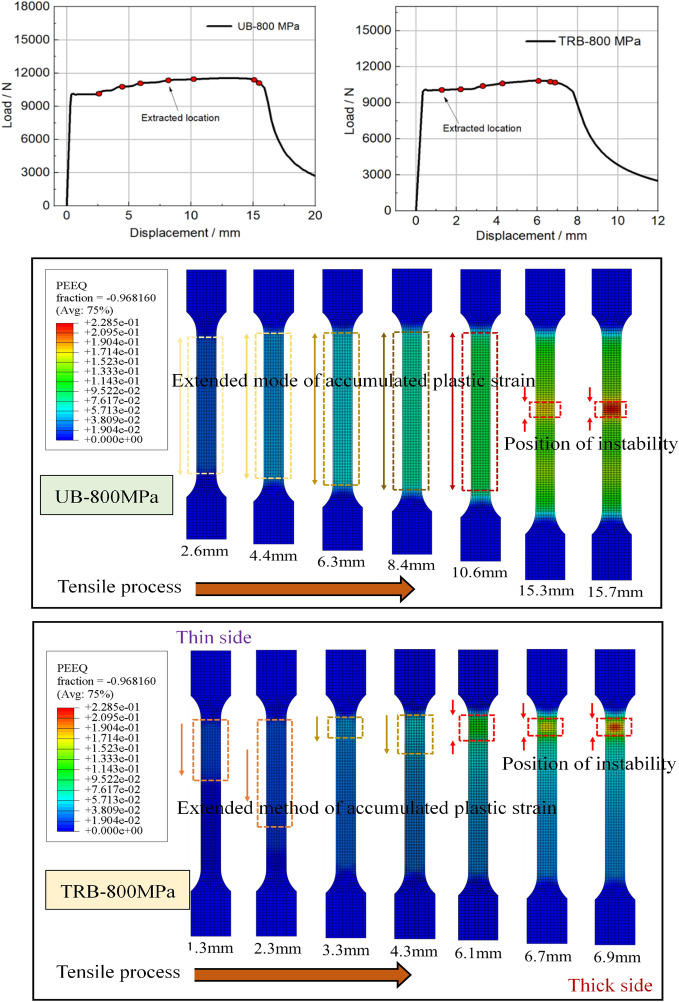
Equivalent plastic strain cloud diagram of ES-TRB-800 MPa and UB-800 MPa during tensile process.

To understand TRB’s plastic deformation, Fig. 23 displays load–displacement curves and strain diagrams for ES-TRB-800 MPa and UB-800 MPa under unidirectional stretching. ES-TRB-800 MPa and UB-800 MPa achieve uniform elongations of 6.1% and 16.19%, with UB-800 MPa showing a 165% increase. The strain distribution in UB-800 MPa is consistent, while ES-TRB-800 MPa shows uneven deformation from the start. The thin zone in ES-TRB-800 MPa consistently has the highest strain, leading to instability. This non-uniformity increases over time, indicating TRB stretching lacks a clear uniform deformation stage. The material beyond the thin zone in TRB is underutilized due to its higher load-bearing capacity. Different thickness areas in ES-TRB-800 MPa have similar initial load-bearing capacities, but vary in deformation due to differing subsequent capacities. This results in TRB’s limited overall uniform elongation. The TRB sample’s uniform elongation is affected by the clamping head, impacting overall elongation. Improving TRB’s elongation requires adjusting the load-bearing capacities in different thickness areas without changing the geometric structure. Maintaining consistent strength across thicknesses reduces uneven deformation. Plastic instability in UB with uniform properties is unpredictable, while in TRB, it’s predictable due to varying load-bearing capacities across thicknesses.

**Figure 24:**
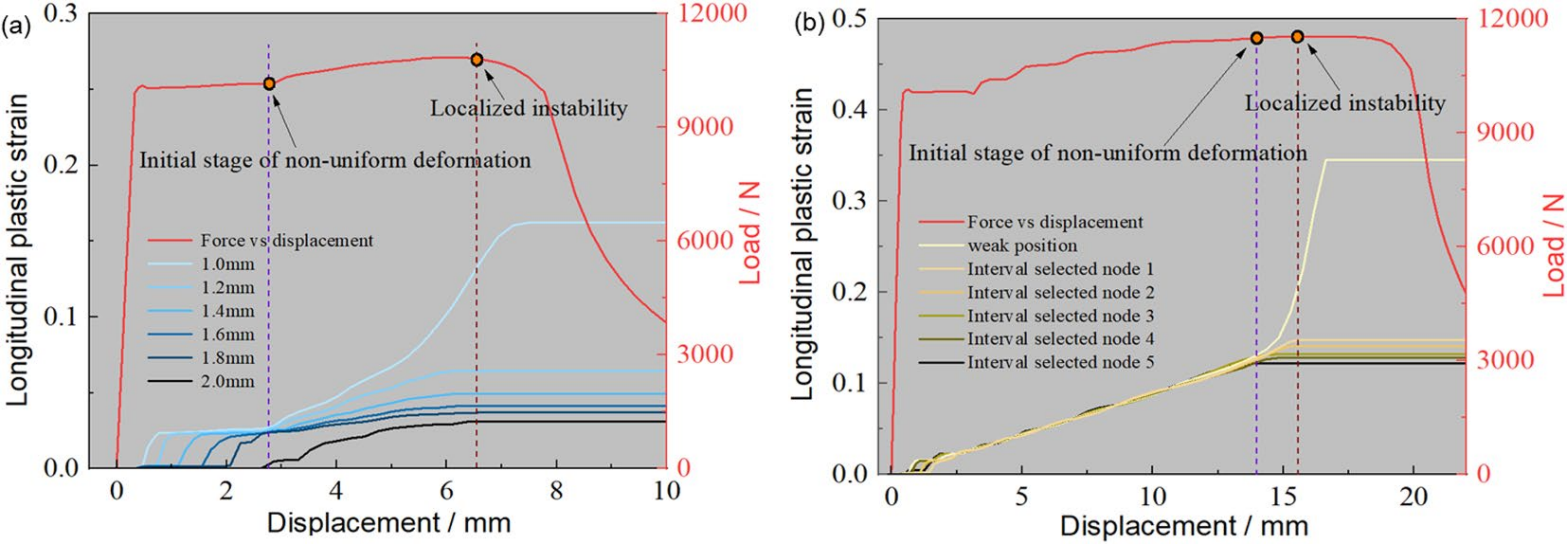
Longitudinal plastic strain in discrete points of ES-TRB-800 MPa and UB-800 MPa. (**a**) ES-TRB-800 MPa; (**b**) UB-800Mpa.

To understand the inhomogeneous deformation mechanism, Fig. 24 shows the longitudinal plastic strain at discrete points across TRB-800 MPa thicknesses. For comparison, the strain in UB-800 MPa’s parallel segment is also provided. Inhomogeneous plastic strain is evident throughout the stretching. Yielding starts first at the thinnest point, then in ascending thickness order. Initial plastic strain is 0.33% at the thinnest point, and the maximum is 2.06% at the thickest. This contrasts with our previous analysis of simultaneous yielding. All thicknesses yield at once due to equal capacity, but it’s subtle. As stretching continues, uniform deformation becomes inhomogeneous due to varying capacities. Deformation disparity increases post-yield stage, meaning thinner areas bear more deformation until maximum load. At peak load, only the thin area elongates, while outer parts stop deforming, resembling a “frozen state”. This highlights TRB’s low plasticity. Plastic instability’s location is predictable due to strain concentration in the thin zone. For UB-800 MPa, mostly uniform deformations are seen, but perfect uniformity is impossible. Different sections enter the plastic zone at varying times, showing uneven deformation from the start. Uniform deformation is achieved through deformation strengthening effects. With equal capacities, deformations are uniform. However, as non-uniform deformation starts, stress instability arises, possibly causing deformation bifurcation and plastic instability, making UB’s instability position prediction challenging.

**Figure 25:**
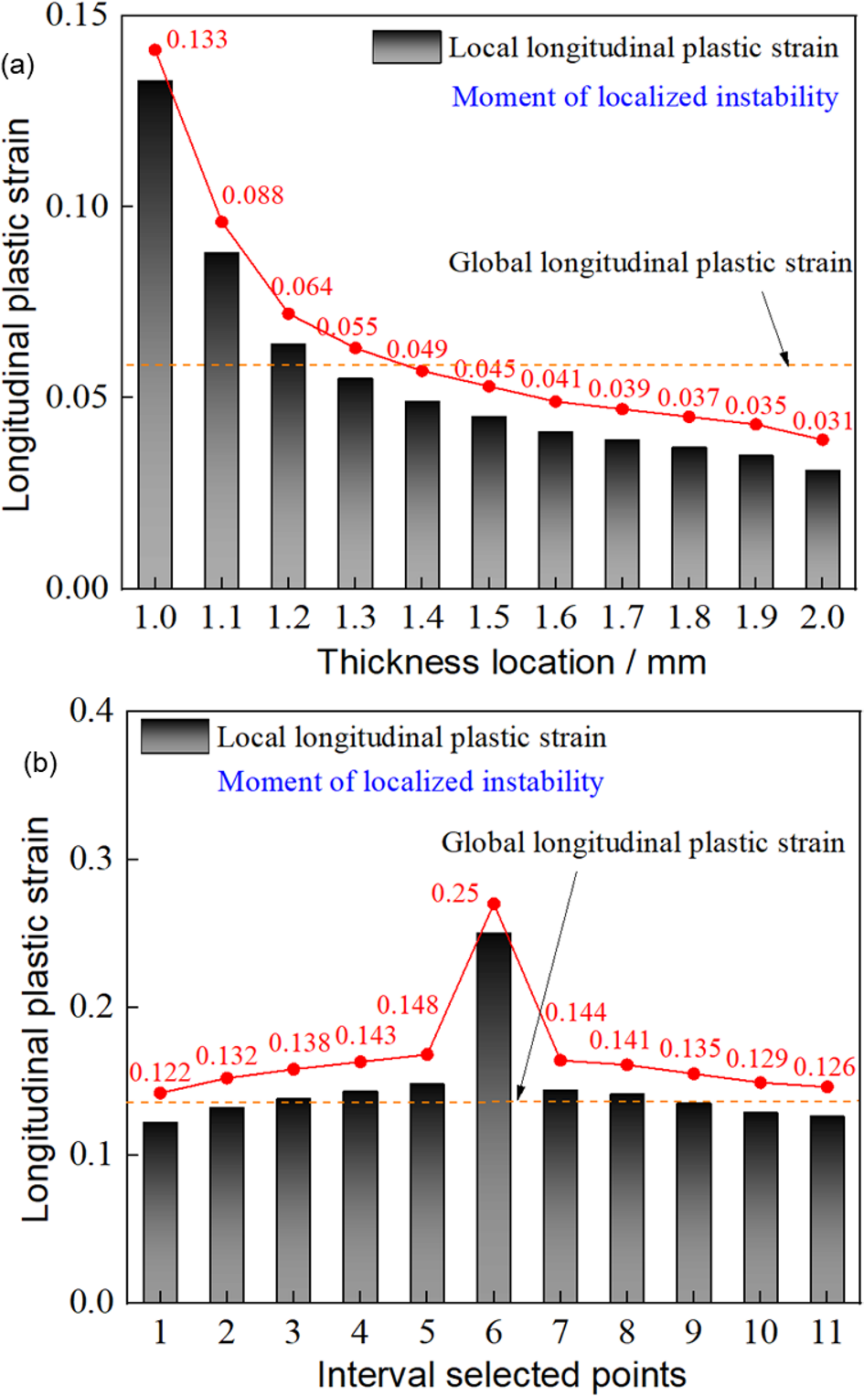
Specific longitudinal plastic strain in different thickness positions of ES-TRB-800 MPa and UB-800 MPa. (**a**) ES-TRB-800 MPa; (**b**) UB-800 MPa.

For a quantitative depiction of longitudinal plastic strain, the longitudinal plastic strain in various discrete selection points of both TRB and UB during plastic instability has been presented in Fig. 25. The analysis reveals that in TRB, local longitudinal plastic strain increases from 0.031 to 0.133 as thickness position rises. This stark non-uniformity in strain distribution indicates that the load-bearing potential of materials beyond the thin zone remains largely untapped. Importantly, it becomes evident that the position of plastic instability for TRB-800 MPa is indeed situated in the thinnest zone. In contrast, the local longitudinal plastic strain of UB ranges from 0.122 to 0.148 outside the plastic instability position. Consequently, a substantial portion of TRB’s load-bearing potential remains unutilized. In terms of overall elongation performance, the global longitudinal plastic strain for TRB and UB is 0.059 and 0.138 respectively. This is nearly identical to the overall uniform elongation, underscoring the point of inadequate carrying potential.

**Figure 26:**
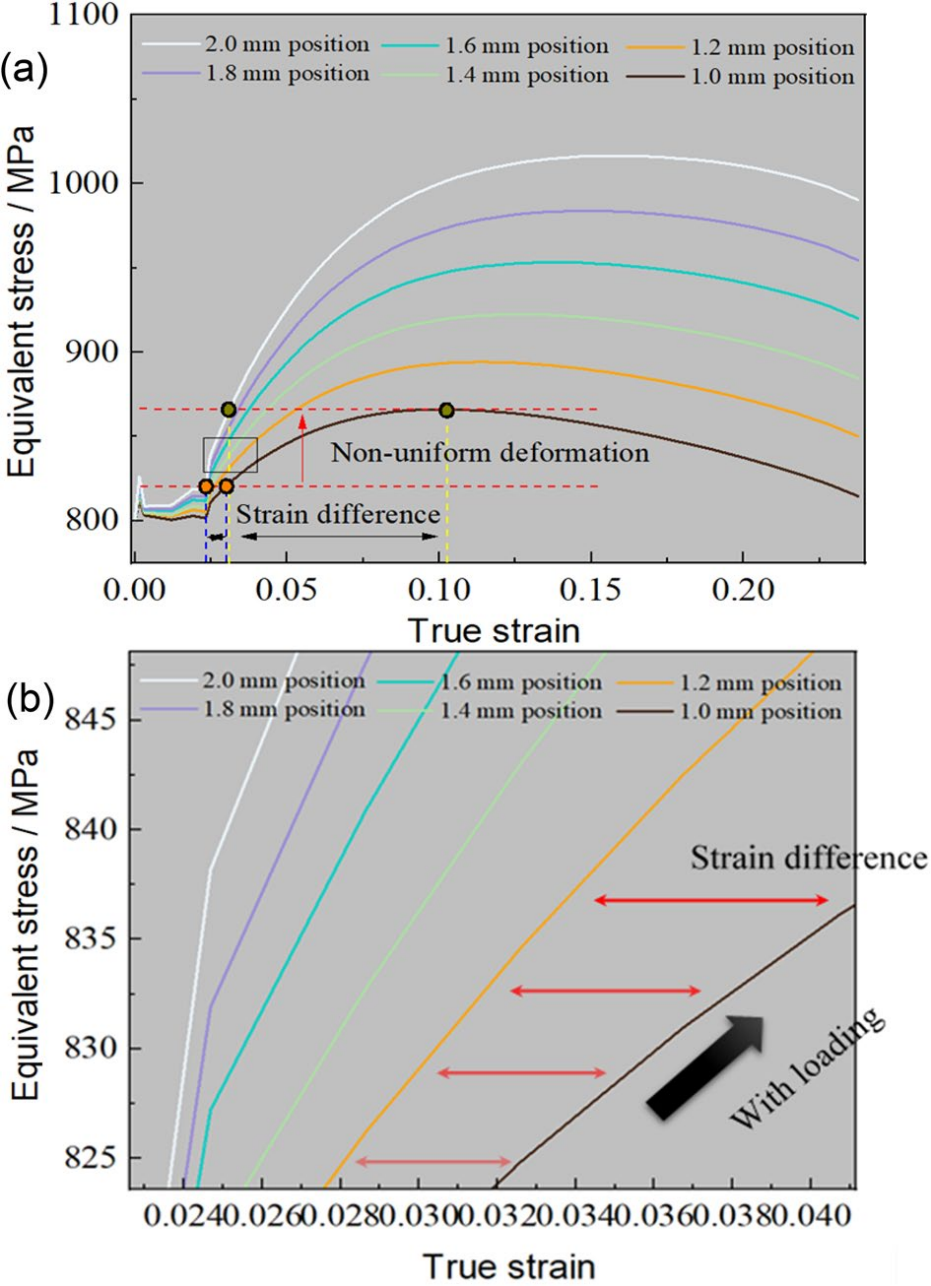
Equivalent stress in different thickness positions of ES-TRB-800 MPa. (**a**) Macroscopic view; (**b**) Magnification of one segment.

Building upon the preceding analysis, the emergence of inhomogeneous plastic deformation primarily stems from the variance in subsequent carrying capacities. Consequently, the equivalent stress (variation of equivalent strength during deformation process) of different thickness positions in TRB-800 MPa has been graphed in Fig. 26. The data illustrates notable disparities in equivalent stress across distinct thickness positions, underscoring substantial discrepancies in carrying capacity. Under the influence of strain hardening, as the external load incrementally increases, varying degrees of deformation are needed to achieve the external load across different thickness positions. Thus, non-uniform deformation manifests initially under equivalent force conditions. While slight strain differences exist among thickness positions in the early stages, their impact remains comparatively limited. However, these strain discrepancies intensify notably upon entering the strain hardening phase. Notably, the thinnest zone boasts the lowest maximum loading capacity, which progressively improves with increasing thickness. When the external load reaches the maximum loading capacity of the thinnest zone, the external force applied to sustain continuous deformation in this region begins to decrease. In contrast, other thickness zones maintain their deformation conditions. Subsequently, the other regions no longer meet the requirements for sustained plastic deformation, transitioning into elastic unloading. Meanwhile, the thinnest region persists in maintaining plastic deformation due to applied external forces, culminating in the concentration of plastic deformation and triggering tensile instability. This aligns with the findings suggesting that plastic instability emerges within the region characterized by the weakest bearing capacity.

The aforementioned phenomenon can also be deduced from a theoretical standpoint. Let’s consider two positions with varying thicknesses from the transition zone of TRB samples. We denote the initial cross-sectional areas before stretching as *A*_01_ and *A*_02_, respectively. At any given moment during the stretching process, the deformation of these areas can be expressed as follows:

1 $$\sigma_{1} A_{1} { = }\sigma_{2} A_{2} ,$$

where *σ*_1_ and* σ*_2_ represent the tensile stress at the two locations with different thicknesses, and *A*_1_ and* A*_2_ represent the real-time cross-sectional areas of the selected positions.

To describe the material’s constitutive equation, the Ludwik equation is employed.

2 $$\sigma { = }H + K\varepsilon^{n} ,$$

where *H*, *K* and *n* denotes the yield strength, hardening coefficient and strain hardening exponent.

Therefore, during the plastic deformation stage, there are:

3 $$\sigma_{1} { = }H_{{1}} { + }K_{{1}} \varepsilon_{1}^{{n_{1} }} ,\sigma_{2} { = }H_{{2}} { + }K_{{2}} \varepsilon_{2}^{{n_{2} }} ,$$

4 $$A_{1} { = }A_{01} e^{{ - \varepsilon_{1} }} ,A_{2} { = }A_{02} e^{{ - \varepsilon_{2} }} .$$

The formulas (3) and (4) have been substituted into formula (7) and reorganized, yielding:

5 $$\frac{{H_{1} { + }K_{1} \varepsilon_{1}^{{n_{1} }} }}{{H_{2} { + }K_{2} \varepsilon_{2}^{{n_{2} }} }} = \frac{{A_{02} e^{{ - \varepsilon_{2} }} }}{{A_{01} e^{{ - \varepsilon_{1} }} }}.$$

If the ratio of cross-sectional areas between two thickness positions is defined as:

6 $$a_{0} = \frac{{A_{02} }}{{A_{01} }},$$

7 $$\frac{{H_{1} { + }K_{1} \varepsilon_{1}^{{n_{1} }} }}{{H_{2} { + }K_{2} \varepsilon_{2}^{{n_{2} }} }} = a_{0} e^{{\varepsilon_{1} - \varepsilon_{2} }} .$$

If the strain ratio between these two thickness positions during the tensile process is defined as:

8 $$m = \frac{{\varepsilon_{2} }}{{\varepsilon_{1} }}.$$

Then the formula (7) can be rearranged as:

9 $$\varepsilon_{1} { = }\frac{{\ln \frac{{H_{1} { + }K_{1} \varepsilon_{1}^{{n_{1} }} }}{{a_{0} (H_{2} { + }K_{2} (m\varepsilon_{1} )^{{n_{2} }} )}}}}{1 - m}.$$

If the Hollomon equation is adopted to describe the material constitutive equation, then the stress–strain relationship can be expressed as:

10 $$\sigma { = }K\varepsilon^{n} .$$

Meanwhile, if the hardening coefficient and strain hardening exponent of these two thickness positions are defined as:

11 $$\kappa = \frac{{K_{2} }}{{K_{1} }},n = \frac{{n_{2} }}{{n_{1} }}.$$

Then formula (9) can be converted to the following expression based on the aforementioned process:

12 $$\varepsilon_{1} { = }\frac{{\varepsilon_{1}^{{n_{1} - n \cdot n_{1} }} \cdot m^{{ - n \cdot n_{1} }} - \ln \kappa a}}{1 - m}.$$

By substituting the remaining parameters into formulas (9) and (12), the strain ratio between the selected two thickness positions can be calculated for the entire tensile process. It is proved that the strain ratio gradually increases as specific parameters are applied. This theoretical explanation aligns with the observed development pattern of non-uniform deformation in TRB.

**Figure 27:**
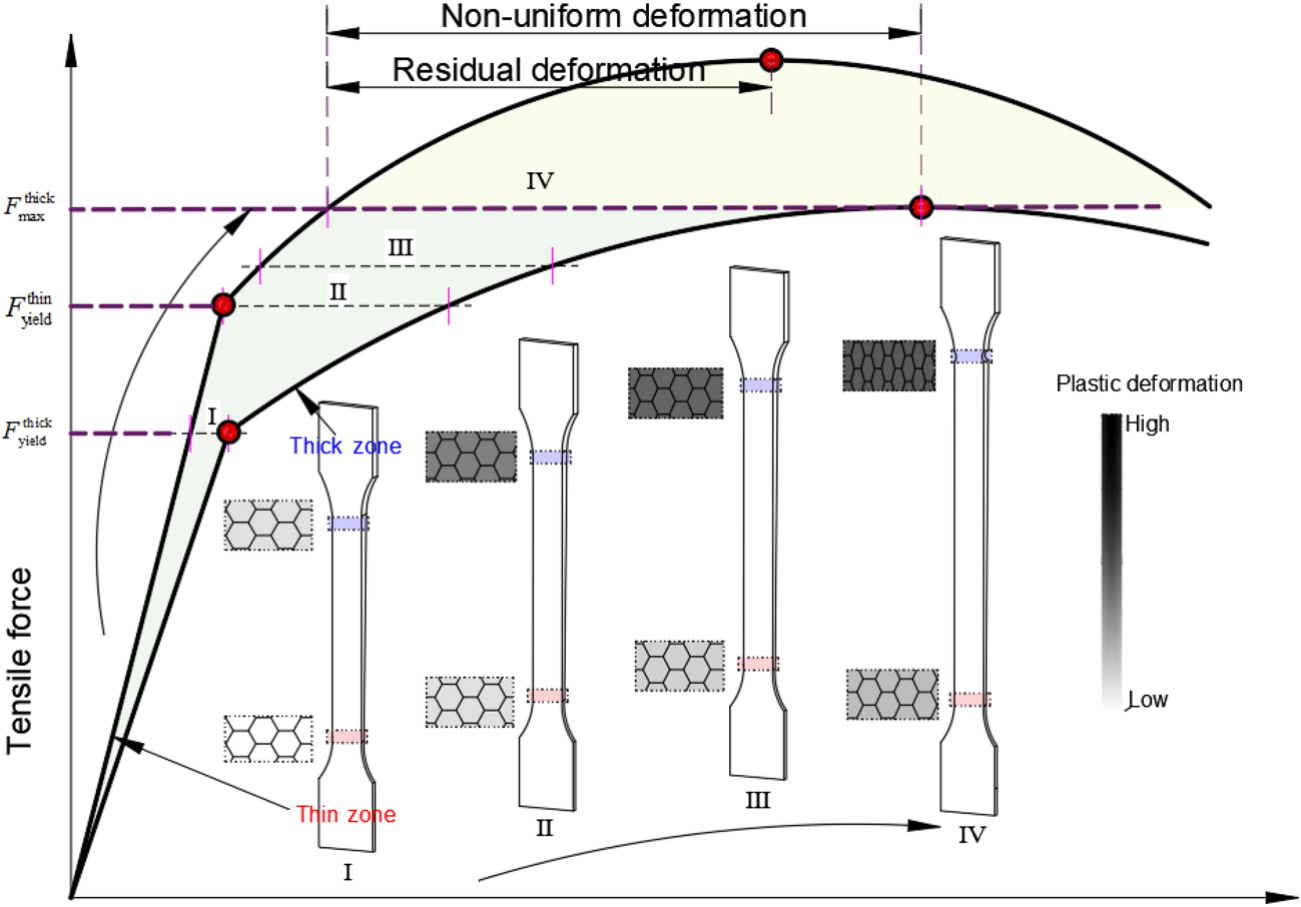
Mechanism diagram of stretching process for TRBs with various mechanical property distributions.

To provide a clearer understanding of the location and cause of plastic instability in TRB, Fig. 27 presents a mechanism diagram of the stretching process. The primary reason for the uneven deformation is the disparity in equivalent stress or load-bearing capacity during stretching. It’s important to note that the mechanical property distribution of TRB can be adjusted using tailor heat treatment technology while maintaining an unchanged thickness distribution. Consequently, the location of plastic instability may not solely reside in the thin zone; rather, plastic instability could occur initially in any position where the ultimate equivalent stress is the lowest. As depicted in Fig. 27, a lower ultimate bearing capacity within a zone result in greater plastic deformation, potentially leading to a swift entry into the plastic instability stage. However, it’s worth highlighting that the position with the lowest equivalent stress might not always lead to plastic instability. In cases where the ultimate equivalent stress is equal or close across different thickness regions, the location with the lowest uniform elongation will dictate the occurrence of plastic instability. Based on this analysis, two design directions can be considered. Firstly, tailor heat treatment regulation can be employed to ensure the consistency of constitutive equations across different thickness positions. This approach can significantly enhance the uniform elongation performance of TRBs, thereby improving subsequent forming capabilities. Secondly, deliberate design and control of low equivalent stress in specific local positions can induce plastic instability at designated locations while preventing it from occurring elsewhere. This insight provides theoretical underpinning for enhancing the design safety of TRBs and TRB structures.

### Axial loading of TRT

Engineering structures use buckling to absorb and dissipate energy during collisions, protecting passengers and vital components. Thin-walled tubes, due to their properties, have higher axial stiffness and resistance to deformation. Thus, the axial collapse of these tubes is a research focus. They collapse in specific modes under axial loading, based on their dimensions and properties. To meet safety standards, it’s essential to control their deformation sequence and initial acceleration. The initial buckling position significantly affects these factors. This section examines the buckling behavior of Tailor Rolled Tubes (TRTs) and Uniform Thickness Tubes (UTs) under axial loading, considering various strength distributions.

#### FE model for axial crushing of TRT

**Figure 28:**
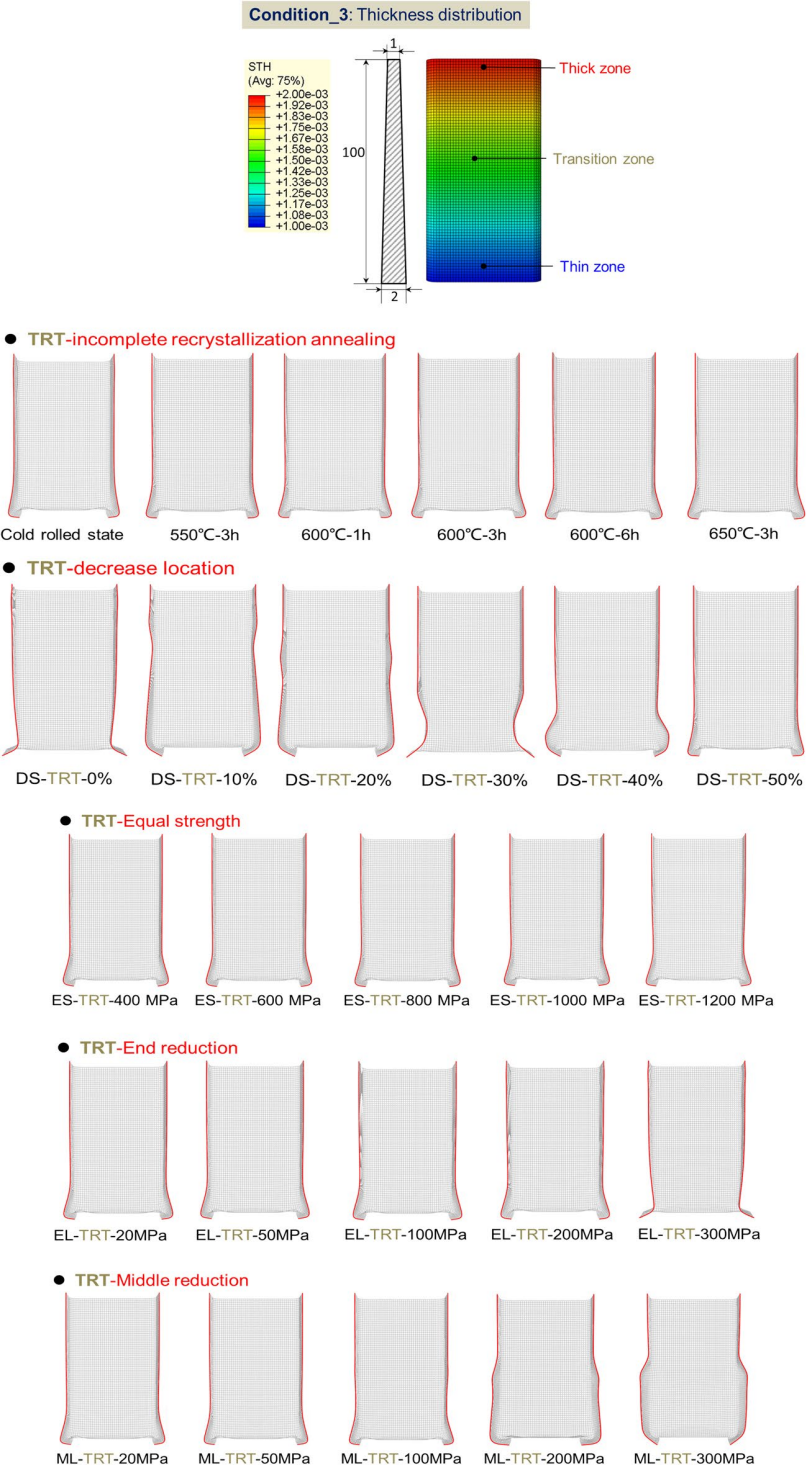
Buckling deformation form of section of TRTs with different mechanical property distributions.

**Figure 29:**
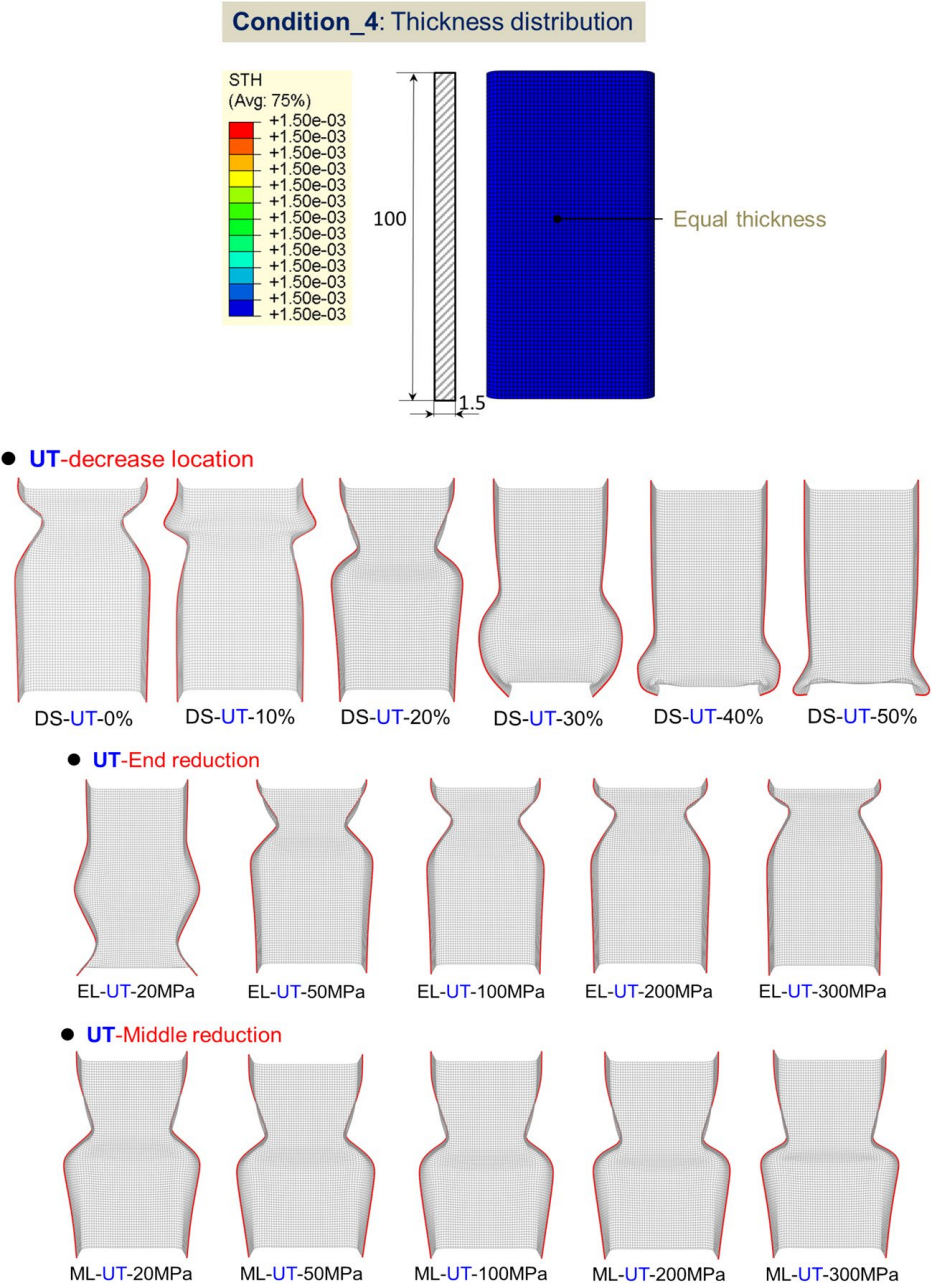
Buckling deformation form of section of UTs with different mechanical property distributions.

**Figure 30:**
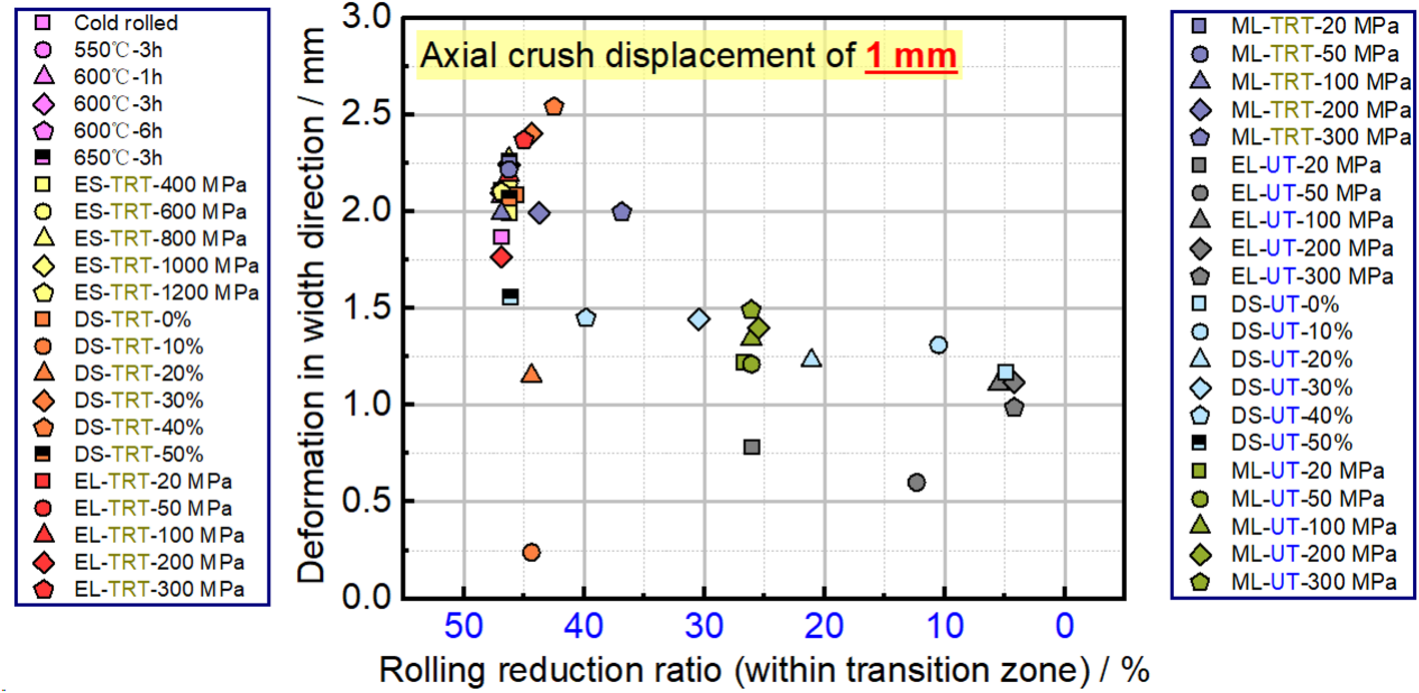
Deformation of the central initial plastic buckling and its rolling reduction ratio in TRTs and UTs.

The behavior of TRTs and UTs under quasi-static conditions is simulated using ABQUS/CAE 2021. Figures 28, 29 and 30 show the geometric configurations of TRTs and UTs. In the simulation, tubes are fixed at the bottom and compressed from one end at 100 mm/s. Four-node shell elements (S4R) with 9 integration points are used for modeling, with a global element size of 1 mm. A mass scaling factor of 100 reduces computational time. Surface interactions use a penalty friction formulation with a friction coefficient of 0.2, and self-contact uses a hard contact model. Tubes have assigned distributions of thickness and mechanical properties. Key properties include density (ρ) = 7850 kg/m^3^, elastic modulus (E) = 210 GPa, and Poisson’s ratio (ν) = 0.33. The model’s accuracy is validated against experimental results^38^. The model predicts TRTs’ deformation and mechanical response. Mechanical property distributions in Figs. 11, 17 and 19 are assigned to study buckling behaviors. It’s noted that buckling simulations have different force conditions than tensile samples of TRB.

#### Effect of mechanical property distributions on plastic instability of TRTs

Figures 28 and 29 show the buckling deformation of TRTs and UTs with varying mechanical properties under specific displacements. The dimensions of TRTs and UTs are set at 100 mm, 50 mm, and 5 mm for height, cross-section, and corner fillet, respectively. An axial displacement of 8 mm is applied to study the effect of strength distribution on structural instability. Thin-walled tubes can experience instability under axial loading, transitioning from load-bearing to local buckling as they near their strength limit. TRTs with incomplete recrystallization show initial plastic buckling near the thin zone due to minor strength differences in the cold rolled state. The position of plastic buckling and deformation is influenced by strength distribution. TRTs with different reduction locations show varied buckling behavior. Classical buckling theory suggests buckling height increases with thickness and strength, leading to overall deformation in thicker zones. TRTs with uniform strength show buckling only in the thin zone, similar to tensile TRB samples. TRTs with reduced strength in the thick zone primarily buckle near the thin zone, but this shifts with strength reduction levels. UTs with strength reduction at one end buckle near that end, highlighting the sensitivity of buckling position to strength in UTs versus TRTs. For TRTs with middle zone strength reductions, buckling occurs in the thin zone for reductions under 100 MPa but encompasses the middle and thin zones for reductions over 200 MPa. UTs with middle zone reductions show buckling near the middle for reductions below 100 MPa and in the middle for reductions over 200 MPa, indicating buckling is more likely at the ends.

The initial peak load is vital for assessing energy absorbers’ peak acceleration during impacts. A lower value suggests better impact acceleration reduction. This load is tied to the initial buckling element’s behavior. Specifically, when initial plastic buckling’s central region is nearer to the thinnest end, the peak load is typically lower. Figure 30 shows the deformation of this central region in relation to the rolling reduction ratio for thin-walled tubes. The designed property distributions cause TRTs to deform more in the thin zone, while UTs have varied buckling positions. This makes TRTs better suited as energy absorbers. Initial buckling often focuses near the tube ends due to end friction affecting local force distribution, encouraging buckling there. However, this observation doesn’t fully capture the initial buckling behavior.

**Figure 31:**
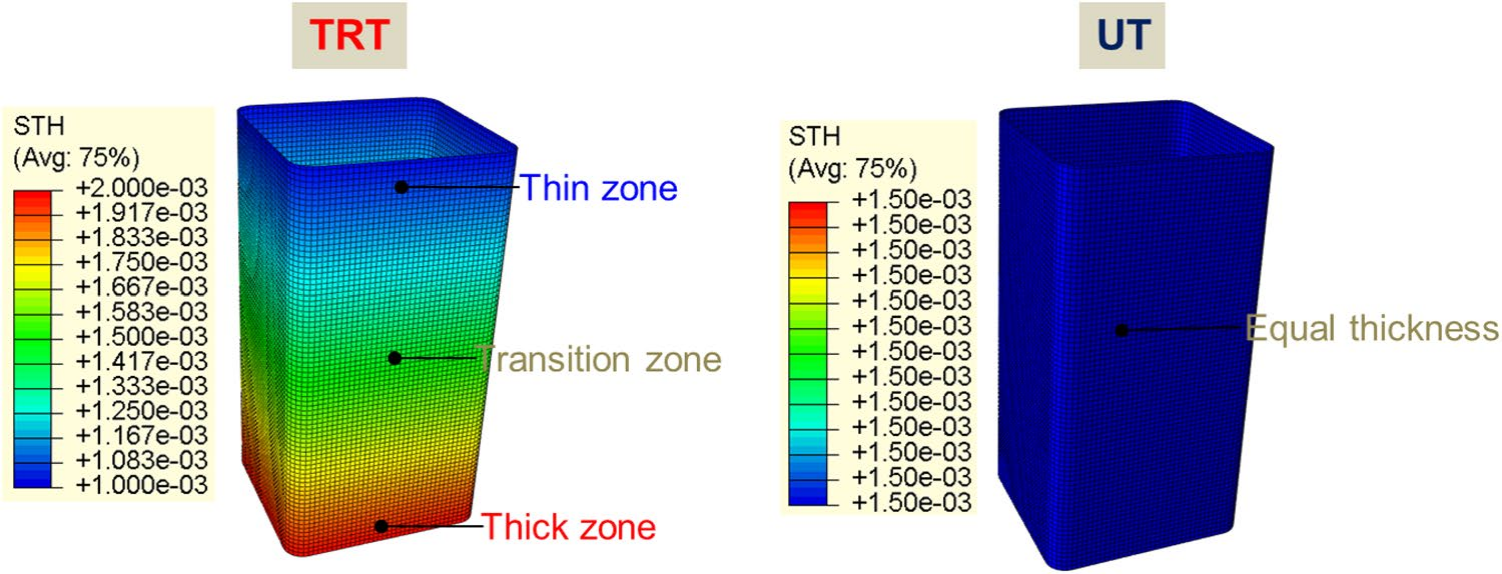
Laying out of TRTs and UTs when adopting FEM.

**Figure 32:**
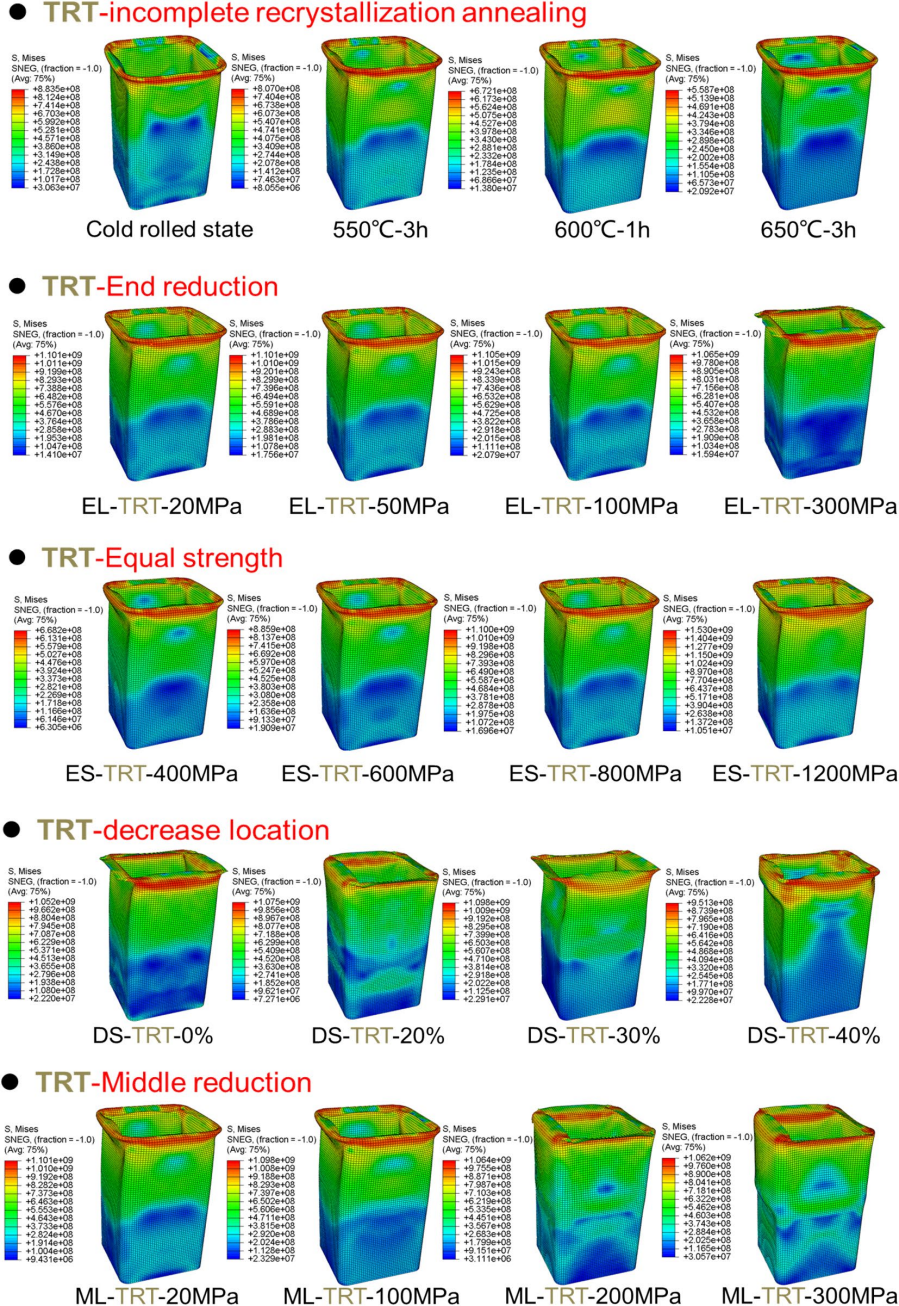
Mises stress distribution of TRTs with different mechanical property distributions under axial loading.

**Figure 33:**
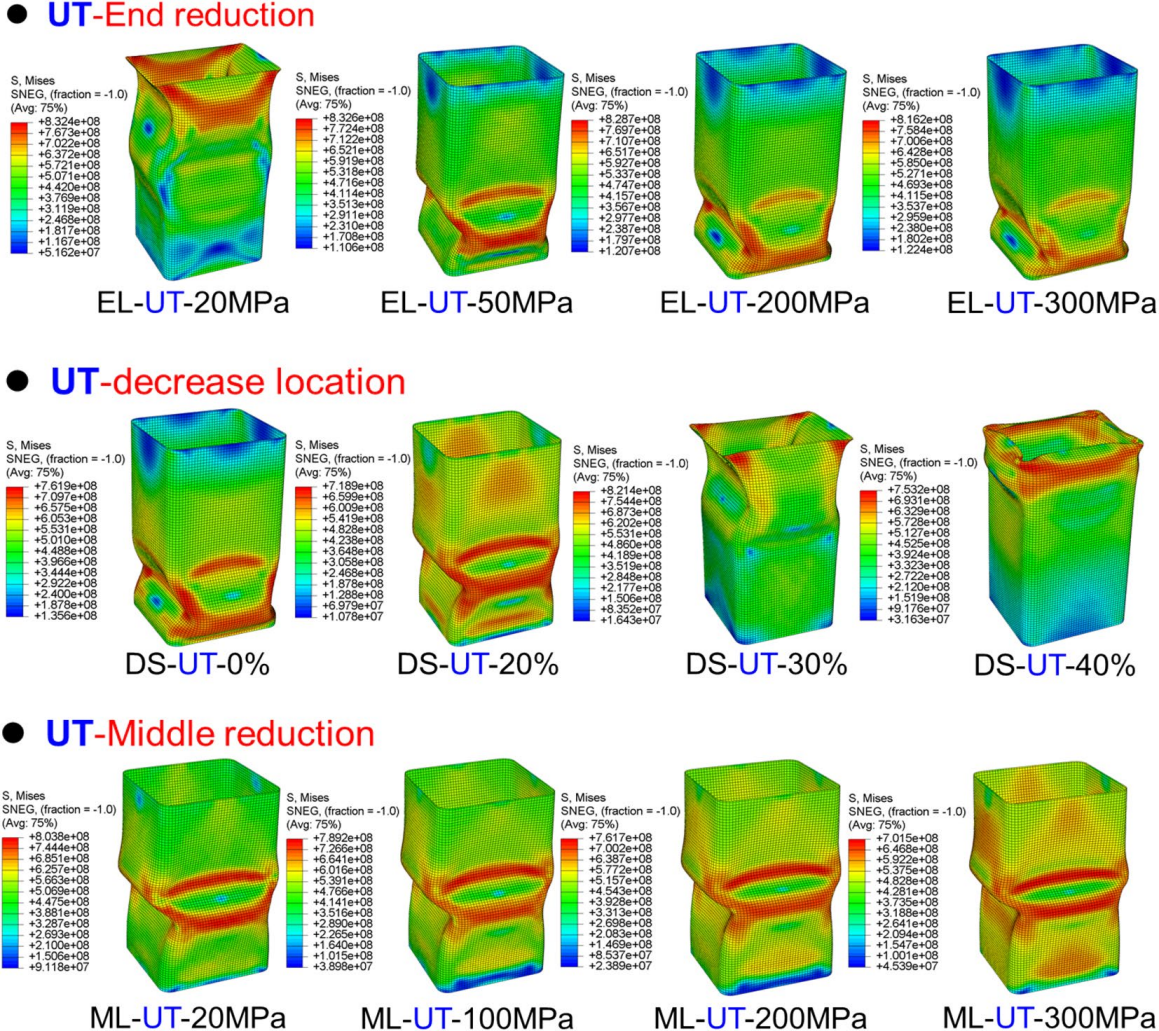
Mises stress distribution of UTs with different mechanical property distributions under axial loading.

The Mises stress distribution of TRTs and UTs with different mechanical property distributions under axial loading is depicted in Figs. 31, 32 and 33, with their corresponding layouts shown in Fig. 31. Specifically, the complete formation of the first buckling element occurs when the compressive displacement reaches approximately 8 mm. Consequently, the Mises stress distributions of TRTs at a displacement of 8 mm are compiled for meaningful comparison. The dimensions of the TRTs and UTs, including height, cross-section dimension, and corner fillet, are uniformly set at 100 mm, 50 mm, and 5 mm. Notably, the height of the tube structures matches the length of the longitudinal transition zone of TRB. To facilitate analysis, a selection of results representing typical characteristics is presented to elucidate differences.

Mechanical property distributions greatly impact the buckling, stress, and deformation of thin-walled tubes. For TRTs with incomplete recrystallization annealing, stress decreases with increased annealing conditions, concentrating in the buckling element’s plastic hinge. Despite local buckling in the thick zone, stress and deformation are more pronounced in the thin zone, especially as buckling develops there. TRTs with end strength reductions can exhibit global buckling in the thick zone, shifting stress away from the thin zone. Differences in stress distribution appear for strength reductions below 50 MPa, with enhanced coordination between zones for reductions over 200 MPa. This coordination is prominent during initial buckling in the thin zone. As strength increases, stress distribution improves, with deformation direction changing for strengths over 1200 MPa, indicating buckling influence expanding to the thick zone. Buckling modes and stress patterns vary with strength reduction location. The distance between buckling elements in the thin zone and reduction locations correlates with strength reduction location. When located in the thick zone, initial separate stress distributions merge, leading to multiple buckling modes. For TRTs with middle zone strength reductions, buckling modes change at a 200 MPa reduction. Below this value, the thin zone is weaker, but above it, buckling occurs in both the middle and thin zones, resulting in a wider stress distribution.

For the UB with end strength reduction, the lower part is highlighted in Fig. 33. Initial buckling appears near the reduction end, with a taller height than TRT structures. As end strength decreases, stress levels drop, indicating that adjusting end strength can control buckling position and peak load. Instability points are mainly at regions with a 200 MPa strength reduction in both TRT and UT samples. For UTs with middle strength reduction, buckling is evident near the middle. Stress focuses in the middle and adjacent end, intensifying in the middle for the latter. Lateral constraints cause end zone deformation when middle strength reduction is low. With sufficient middle reduction, buckling occurs there. Stress concentration above the middle is due to ample deformation space. Adjusting properties away from the end can induce buckling, but the needed reduction is higher than for the end condition.

**Figure 34:**
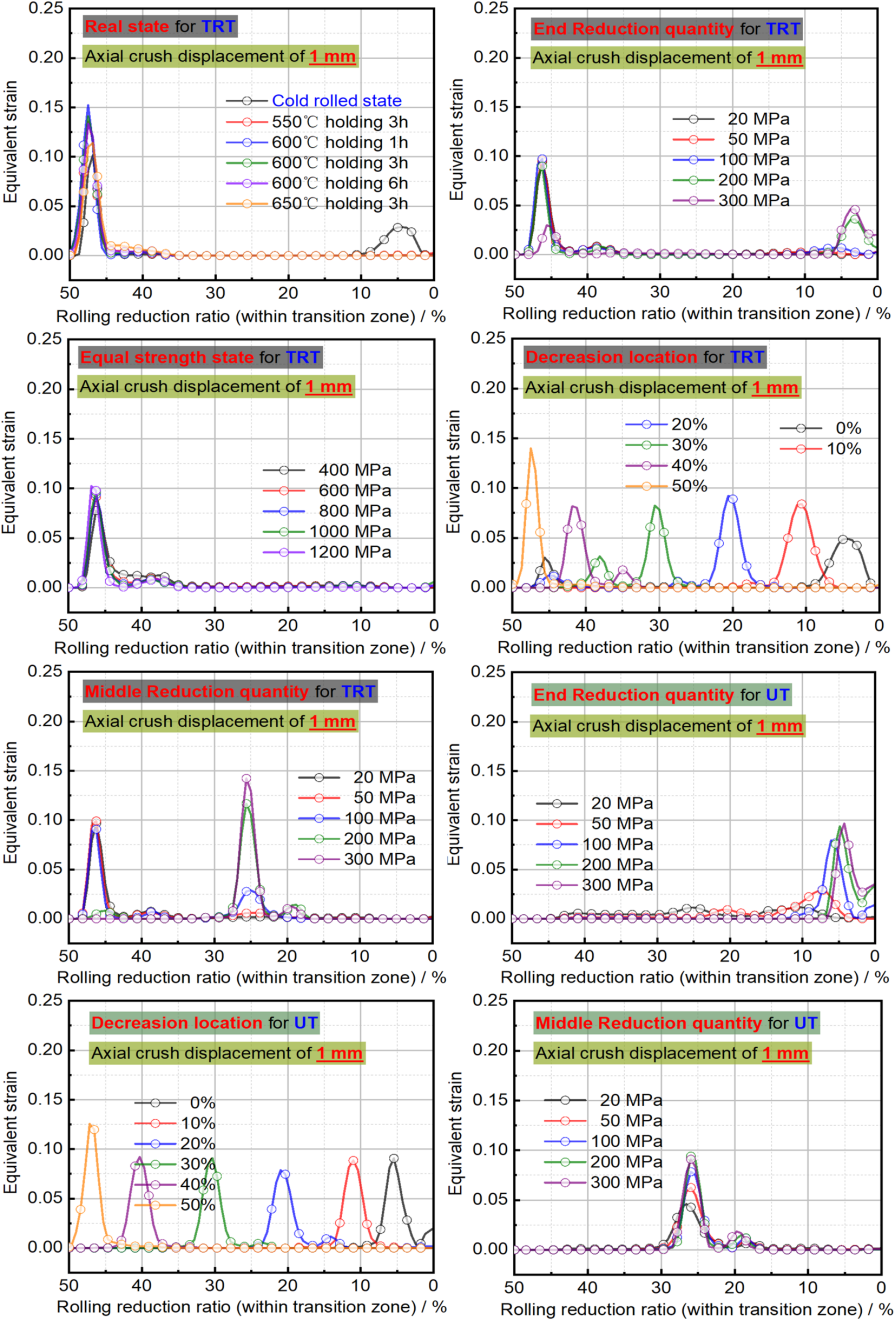
The equivalent strain distribution of TRTs and UTs with different equivalent strength distributions.

Figure 34 shows the strain distribution in TRTs and UTs with different strength distributions. For TRTs with incomplete recrystallization annealing, strains mainly appear in the thin end zone, aligning with the buckling element’s central hinge. Under cold rolled conditions, two peak strains emerge due to similar strength values, causing extended strain coordination. TRTs with end strength reduction show strain near the thin zone’s reduction end, especially when reduction is under 200 MPa. At 300 MPa reduction, strain shifts to the opposite end with a secondary peak strain exceeding the thin zone’s strain. This indicates that plastic buckling starts with deformation processes, with stress concentration aiding the deformation shift from thick to thin zones. TRTs under axial loading are less sensitive to end reduction than tensile TRBs. TRTs with uniform strength have strain patterns like tensile TRBs, with a peak near the thin end. TRTs with varied strength reduction locations have major strain in those areas, affecting initial deformation. However, buckling mainly happens near the thin zone, showing that yield strength-based strength reduction primarily determines initial deformation. TRTs with middle strength reduction show major strain near the thin end for reductions under 100 MPa, moving to the middle at 200 MPa. This differs from tensile TRBs due to end restrictions. Axial loading in TRTs results in diverse strain patterns affected by end restriction, load-bearing, and deformation mode. Stress and strain compatibility in TRTs is stronger than in tensile TRBs, meaning specific buckling locations need more strength reduction. Minimal or no reduction initiates buckling at the end. These insights focus on initial buckling, not total energy absorption.

#### Discussion for the structure instability of TRT

**Figure 35:**
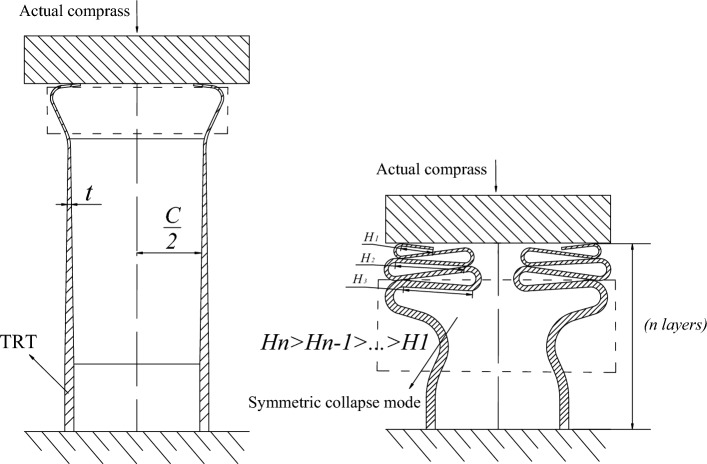
Deformation mechanism of the TRTs under crashing process.

Due to variations in operational conditions, direct application of equivalent strength for determining the position of plastic instability might not yield accurate results, potentially leading to significant discrepancies between predicted and actual plastic instability locations. The deformation mechanism of TRTs during the crash process is illustrated in Fig. 35. Building upon prior research by the author, the parameters of effective material strength (${\sigma }_{t}^{e}$) and the effective thickness of the nth layer (${t}_{n}^{e}$) both depend on the parameter *H*. As a result, ${M}_{n}^{e}$ can also be expressed as a function of *H*. It becomes evident that the crashing force (${P}_{n}$) for TRTs is

13 $$P_{n} = M_{n}^{e} (H) \cdot F(H) = \varphi (t,\sigma_{t}^{e} ).$$

The function *F(H, r)* of this unknown parameter is

14 $$F(H,r) = C_{1} \frac{r}{t} + C_{2} \frac{C}{H} + C_{3} \frac{H}{r},$$

where the parameters of ${C}_{1}, {C}_{2}, {C}_{3}$ are 18.56, 12.57, 8.91.

As a result, the effective yield bending moment per unit length (${M}_{n}^{e}$) can be known as.

15 $$M_{n}^{e} = \frac{{\sigma_{t}^{e} (t_{n}^{e} )^{2} }}{4}.$$

Based on the buckling element’s deformation mechanism, it becomes evident that the bending moment of the buckling element can serve as a predictive indicator of the resistant capacity of TRTs.

**Figure 36:**
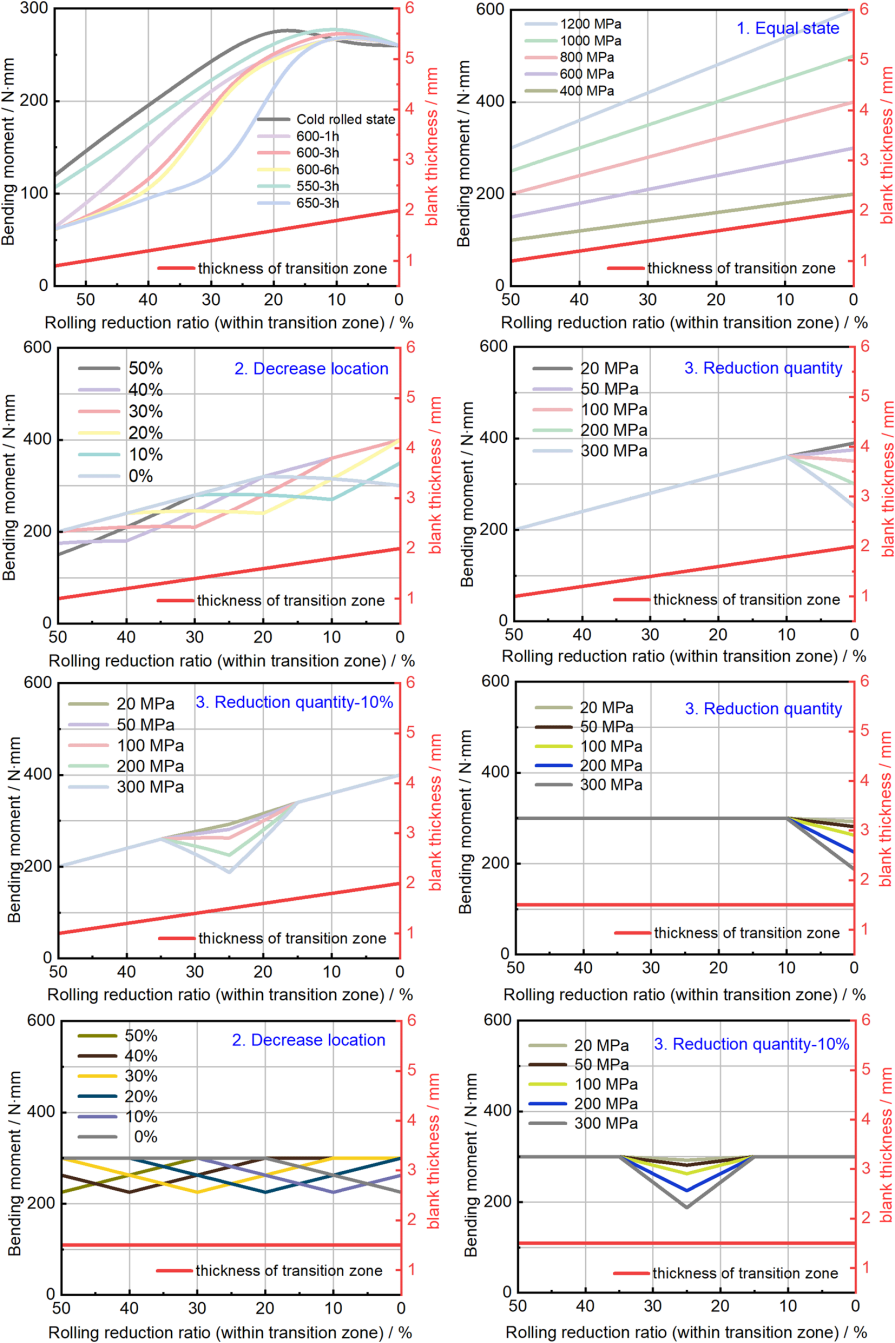
Bending moment distributions across the transition zone of TRT in thickness variation directions.

Consequently, the distribution of bending moments across the thickness variations in TRTs has been compiled in Fig. 36. Notably, our focus should be on the position exhibiting the lowest bending moment within TRTs. Unlike the stretching process, the impact of thickness on the bending moment is amplified twofold during axial crushing, signifying a heightened significance of thickness variation. Predicting the plastic instability of UTs with diverse mechanical property distributions appears comparatively straightforward, as the thickness distribution remains constant. A somewhat more intricate approach involves the simultaneous calculation of two changing factors—thickness and mechanical properties.

The accuracy of these predictions is assessed by comparing the lowest bending moment position and the actual buckling position of TRTs and UTs, along with other key parameters, as summarized in Table 1. Notably, the method of utilizing the lowest bending moment proves more precise for UTs with varied mechanical property distributions, given their relatively minor disturbances. The prediction error rate remains below approximately 12%. However, certain sample predictions exhibit less accuracy, such as ML-TRT-300 MPa, EL-UT-20 MPa, and EL-UT-50 MPa. By referring to Fig. 36, three main factors contributing to this discrepancy are identified. Firstly, when the lowest bending moment is closely situated to the next lowest moment, the internal force may be inadequate to trigger plastic instability. Secondly, divergent boundary conditions at the ends compared to the middle part could counteract the plastic deformation tendency at the position with the lowest bending moment. Lastly, the lower position and range of bending moments, coupled with the distance between them, can influence the plastic instability position. Hence, ensuring that the desired bending moment position possesses sufficient disturbance or gradient becomes essential. Achieving this requires the application of tailor heat treatment technology, guaranteeing plastic instability at the intended position. This approach allows for flexible adjustments to the deformation sequence of safety components, ultimately ensuring the safety of both passengers and critical components.

**Table 1:** Important parameters of TRTs and UTs under axial loading condition.

Sample number	Buckling position/%	Lowest bending moment position/%	Minimum bending moment value/N∙mm	Maximum bending moment value/N∙mm	Error rate/%	Difference ratio between TRB and TRT/%
TRT-Cold rolled	46.88	50	119.48	284.43	− 6.25	− 5.24
TRT-550°C-3 h	46.25	50	106.52	282.38	− 7.5	− 6.5
TRT-600°C-1 h	46.88	50	63.38	272.44	− 6.25	− 6.24
TRT-600°C-3 h	46.88	50	60.75	279.07	− 6.25	− 5.24
TRT-600°C-6 h	46.88	50	60.95	274.11	− 6.25	− 5.24
TRT-650°C-3 h	46.88	50	61.56	274.93	− 6.25	− 5.24
ES-TRT-400 MPa	46.25	50	100	200	− 7.5	− 3.5
ES-TRT-600 MPa	46.25	50	150	300	− 7.5	− 2.5
ES-TRT-800 MPa	46.25	50	200	400	− 7.5	− 1.5
ES-TRT-1000 MPa	46.25	50	250	500	− 7.5	− 0.5
ES-TRT-1200 MPa	46.88	50	300	600	− 6.25	1.76
DS-TRT-0%	45.3	50	150	400	− 8.75	86.6
DS-TRT-10%	44.38	50	175	400	− 11.25	67.76
DS-TRT-20%	44.38	50	200	400	− 11.25	47.76
DS-TRT-30%	44.38	50	200	400	− 11.25	27.76
DS-TRT-40%	42.5	50	200	350	− 15	4
DS-TRT-50%	46.25	50	200	320	− 7.5	− 6.5
EL-TRT-20 MPa	46.25	50	200	390	− 7.5	− 1.5
EL-TRT-50 MPa	46.25	50	200	375	− 7.5	− 1.5
EL-TRT-100 MPa	46.25	50	200	360	− 7.5	− 1.5
EL-TRT-200 MPa	46.88	50	200	360	− 6.25	91.76
EL-TRT-300 MPa	45	50	200	360	− 10	89
ML-TRT-20 MPa	46.25	50	200	400	− 7.5	− 1.5
ML-TRT-50 MPa	46.25	50	200	400	− 7.5	− 1.5
ML-TRT-100 MPa	46.88	50	200	400	− 6.25	42.76
ML-TRT-200 MPa	43.75	50	200	400	− 12.5	37.5
ML-TRT-300 MPa	36.88	25	187.5	400	23.75	23.76
EL-UT-20 MPa	26	0	292.5	300	52	46
EL-UT-50 MPa	12.33	0	281.25	300	24.65	19.66
EL-UT-100 MPa	5.52	0	262.5	300	11.03	8.04
EL-UT-200 MPa	4.25	0	225	300	8.49	7.5
EL-UT-300 MPa	4.2	0	187.5	300	8.43	7.4
DS-UT-0%	4.89	0	225	300	9.78	7.78
DS-UT-10%	10.51	10	225	300	1.01	1.02
DS-UT-20%	21.08	20	225	300	2.15	2.16
DS-UT-30%	30.46	30	225	300	0.91	0.92
DS-UT-40%	39.84	40	225	300	− 0.32	− 0.32
DS-UT-50%	46.12	50	225	300	− 7.75	− 5.76
ML-UT-20 MPa	26.69	25	292.5	300	3.39	5.38
ML-UT-50 MPa	26.08	25	281.25	300	2.15	2.16
ML-UT-100MPa225	26.08	25	262.5	300	2.15	2.16
ML-UT-200 MPa	25.48	25	225	300	0.97	0.96
ML-UT-300 MPa	26.09	25	187.5	300	2.19	2.18

## Conclusions

Research has delved into the plastic instability and mechanical behaviors of TRB and TRT structures under tensile and axial loads, with comparisons to equal thickness structures. We’ve rigorously studied the influence of equivalent strength distribution levels, reduction locations, and amplitude on deformation patterns. Summarized findings are:

1. HC340LA steel-based TRBs show complex mechanical property distributions. Without annealing, strength decreases from 580 to 340 MPa as reduction ratio goes from 50 to 0%. Annealing initially increases strength, then declines along the longitudinal transition zone. Instability primarily arises in the thinnest zone due to the lowest “equivalent stress” and bending moment. Heat treatment doesn’t sufficiently counteract thin zone deformation.
2. Mechanical strength levels influence instability patterns. Instability is predominantly in the thinnest zone due to its reduced carrying capacity. While initial carrying capacities are consistent across thicknesses, they diverge under tensile loading. Axial loading emphasizes the role of thickness in determining carrying capacity.
3. Under tensile conditions, TRBs and UBs show instability at locations with reduced strength. TRBs require strength reduction in the thick zone to balance disparities, while UBs’ deformation coordination is affected. TRTs and UTs exhibit slight differences in instability due to varied force conditions.
4. Equivalent strength fluctuations impact instability position and response. Reduced strength in thick and middle zones induces instability in those areas. For TRBs, the location of instability shifts based on the reduction value. TRTs show concentrated instability near the thin zone, with middle zone reductions causing extensive plastic buckling.
5. TRB samples with customized thickness and properties exhibit non-uniform deformation from the outset. TRBs can’t fully utilize their carrying capacity due to pronounced non-uniform deformation. The position with the lowest ultimate equivalent strength is prone to plastic instability. For TRTs, factors like end friction and bending moment range are crucial for accurate instability prediction.
6. This study suggests two design guidelines:
  Achieving consistent equivalent strength across thicknesses through tailored heat treatment can improve TRBs’ elongation and forming performance.
  Designing and controlling ultimate equivalent stress or bending moment in specific thickness areas can enhance safety in tailored structures.

## Data Availability

Data generated or analyzed during this study are available upon reasonable request. Interested parties should contact the corresponding author, Associate Professor Rihuan Lu, at the National Engineering Research Center for Equipment and Technology of Cold Rolled Strip, Yanshan University, Qinhuangdao, Hebei, 066004, P.R.China. Email inquiries can be directed to lrh@ysu.edu.cn. Requests for access to data will be considered in line with institutional ethical guidelines and scientific integrity standards.
